# Near-Bottom Hypoxia Impacts Dynamics of Bacterioplankton Assemblage throughout Water Column of the Gulf of Finland (Baltic Sea)

**DOI:** 10.1371/journal.pone.0156147

**Published:** 2016-05-23

**Authors:** Peeter Laas, Elina Šatova, Inga Lips, Urmas Lips, Jaak Simm, Veljo Kisand, Madis Metsis

**Affiliations:** 1 Marine Systems Institute at Tallinn University of Technology, Akadeemia Rd. 15A, 12618, Tallinn, Estonia; 2 Department of Gene Technology, Tallinn University of Technology, Akadeemia tee 15, 12618, Tallinn, Estonia; 3 Institute of Technology at University of Tartu, Nooruse 1, 50411, Tartu, Estonia; 4 Institute of Mathematics and Natural Sciences, Tallinn University, Narva Rd. 25, 10120, Tallinn, Estonia; INRA, FRANCE

## Abstract

Over the past century the spread of hypoxia in the Baltic Sea has been drastic, reaching its ‘arm’ into the easternmost sub-basin, the Gulf of Finland. The hydrographic and climatological properties of the gulf offer a broad suite of discrete niches for microbial communities. The current study explores spatiotemporal dynamics of bacterioplankton community in the Gulf of Finland using massively parallel sequencing of 16S rRNA fragments obtained by amplifying community DNA from spring to autumn period. The presence of redoxcline and drastic seasonal changes make spatiotemporal dynamics of bacterioplankton community composition (BCC) and abundances in such estuary remarkably complex. To the best of our knowledge, this is the first study that analyses spatiotemporal dynamics of BCC in relation to phytoplankton bloom throughout the water column (and redoxcline), not only at the surface layer. We conclude that capability to survive (or benefit from) shifts between oxic and hypoxic conditions is vital adaptation for bacteria to thrive in such environments. Our results contribute to the understanding of emerging patterns in BCCs that occupy hydrographically similar estuaries dispersed all over the world, and we suggest the presence of a global redox- and salinity-driven metacommunity. These results have important implications for understanding long-term ecological and biogeochemical impacts of hypoxia expansion in the Baltic Sea (and similar ecosystems), as well as global biogeography of bacteria specialized inhabiting similar ecosystems.

## Introduction

Two of the most significant issues facing coastal zone management are habitat loss and alteration due to anthropogenic pollution fuelled by eutrophication, which in the case of the Baltic Sea is amended with vicious positive feedback loop caused by oxygen depletion leading to increased phosphate release from sediments [1]. Oxygen minimum zones have been expanding on a global scale during the geologically recent deglaciation [2]. There has been about 10-fold increase of hypoxia in the Baltic Sea during past 115 years [3]. Depletion of oxygen alters ecosystem energy flow from macrobenthic organisms to microbes [4]. It is estimated that due to hypoxia about 30% of total secondary production is lost in the area [5], and these losses are expected to increase with the expansion of the oxygen-depleted area.

Estuaries represent the interface between marine and freshwater habitats hosting unique and mixed communities of microorganisms. Spatiotemporal studies of variability of bacterioplankton community composition (BCC) in coastal margins have shown that the spatial variability can extend over seasonal changes, given a sharp gradient of environmental factor (e.g. salinity, oxygen) [6, 7]. A recent study of spatiotemporal dynamics of artificially oxygenated fjord (Byfjord, Sweden) that has comparable trophic conditions with the Baltic Sea demonstrated that the amount of oxygen available shaped the bacterial communities, regardless of the depths or the season they were collected in [7].

The presence of redox zone, and thereby different chemical gradients of electron donors and acceptors in variable limiting conditions results in redox-driven niche partitioning. Such geographically distant oxygen minimum zones can be inhabited by related bacterial lineages [8, 9]. A previous spatiotemporal investigation in the Gulf of Finland showed that the main factor shaping the BCC is not only the redox-driven niche partitioning, but ‘key species’ are indeed closely affiliated with bacterial sequences isolated from other similar oxygen depleted ecosystems.

Vertical and/or horizontal surveys of the Baltic Sea have demonstrated that various environmental gradients have a profound impact on BCC [10–19] and cause zonation of functional capabilities [20, 21]. However in temperate climates dramatic seasonal shifts and annually repeating patterns in the BCC should not be overlooked [22–27]. Though, datasets restricted to single dimensions, such as time series from single station survey, can be hard to analyse in order to distinguish between arbitrary shifts in water masses and actual biological succession in bacterioplankton communities driven by changes in environmental condition [24]. The present investigation was undertaken to breach this limitation.

Here we present a study that compares BCC in multiple dimensions: two spatial scales (vertical and horizontal variation) and time. For this purpose massively parallel sequencing of amplified 16S rRNA gene fragment libraries was performed. Samples were collected from two transects in the Gulf of Finland from spring to autumn in 2012. The goal of the present investigation is to characterize spatiotemporal patterns of microbial community composition in hydrographically complex Gulf of Finland that is also the sub-basin to where the oxygen depleted zone is expanding from the Baltic Proper.

## Materials and Methods

### Ethics statement

No specific permits were required for the described field studies. The location is not privately-owned or protected in any way. The field studies did not involve endangered or protected species.

### Study area and sample collection

**Fig 1:**
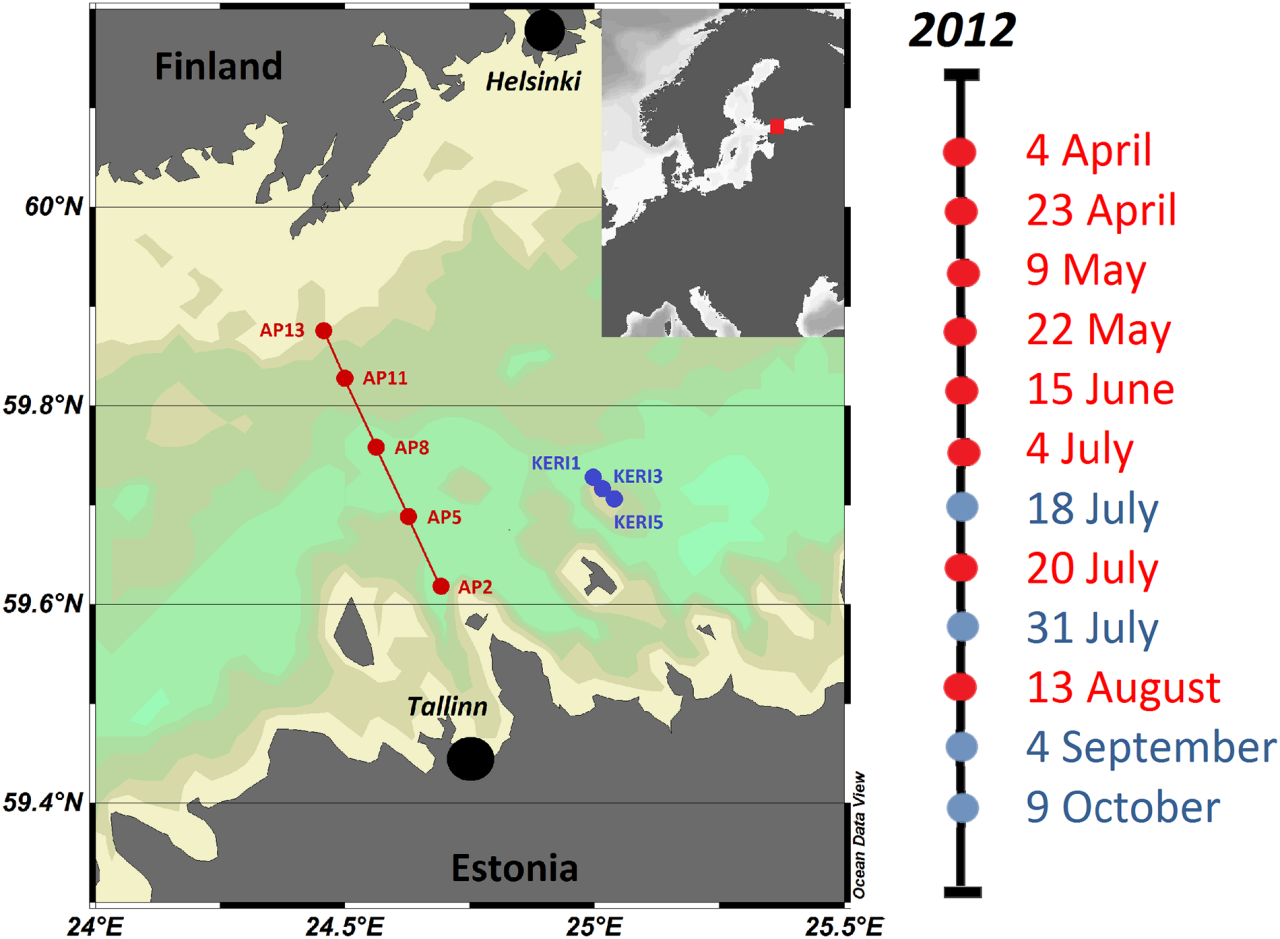
Map of central Gulf of Finland supplemented with AP- and KERI-transects, stations are color-coded red and blue, respectively. The timeline of the 2012 sampling cruises is provided with corresponding color-codes. Four samples were also collected on 8th November of 2011. The tan to bright green colours give an indication of the topography of the study area.

Water sampling aboard the R/V Salme was performed on two transects in the central part of the Gulf of Finland from April to October of 2012 (Fig 1). The cross-gulf AP-transect was sampled to characterize south-north salinity gradient maintained by a cyclonic circulation pattern in the gulf. This residual circulation consists of an outflow of fresher waters (originating from the main river discharge at the eastern end of the gulf) in the northern part and an inflow of saltier waters from the Northern Baltic Proper along the southern coast [28]. Vertical stratification of the water column and water exchange with the Northern Baltic Proper are characterized by high variability both in long-term [29] and in monthly scales [30]. The measurements along the Keri-transect were conducted to sample the deepest site in the central gulf where anoxic bottoms could occur with relatively high probability. Samples were collected from three to five depths, depending on the transect (S1 Table). A rosette sampler (M1018, General Oceanics, Inc.) equipped with Niskin water samplers (volume 1.7 l) was used for sampling. For background information the vertical profiles of temperature and dissolved oxygen were obtained with an OS320plus CTD probe (conductivity, temperature, depth probe with oxygen sensor, Idronaut s.r.l) while chlorophyll *a* fluorescence was registered with a Seapoint chlorophyll *a* fluorometer.

### Sample Processing

Water samples for microbial community DNA extraction were collected into sterile bottles (Nalgene, Thermo Scientific Inc.) and immediately filtered on 0.2 μm filters (Puradisc FP 30; Whatman, Inc.) after preliminary filtration through 5.0 μm prefilters (Puradisc FP 30; Whatman, Inc.). The design of filtration system is presented in [17]. The sample volume varied between 0.5 and 1.0 liters. Filters were kept frozen at—20°C until community DNA was extracted with a PowerSoil^®^ DNA Isolation Kit (MO BIO Laboratories, Inc.). Few modifications were made to the protocol: syringe filters were incubated with the lysis buffer in the casing at 60°C for 30 minutes and then eluate was removed. Water samples for flow cytometry were fixed with paraformaldehyde (PFA, 1% final concentration). Cell counts were obtained with Accuri C6 flow cytometer (Becton Dickinson) after being stained with SYBR Green I DNA dye for 15 minutes at room temperature [31].

### Amplification of bacterial 16S rRNA gene sequences

The bacterial 16S rRNA gene V1-V2 hypervariable regions were amplified in polymerase chain reactions (PCR) using universal bacterial primers BSF8 and BSR357 complemented with 8 nt index and Illumina adapter sequences [32, 33]. PCR was performed with Smart-Taq Hot Red 2X PCR Mix (Naxo, Estonia), 1 μl of extracted DNA, 0.2 μM of each primer, using the following cycling parameters: 15 minutes denaturation at 95°C followed by 3 cycles (30 sec at 95°C, 30 sec at 50°C, 60 sec at 72°C), 28 cycles (30 sec at 95°C, 30 sec at 65°C, 60 sec at 72°C) and a final extension at 72°C for 7 minutes. PCR reactions were run in a Thermal cycler 2720 (Applied Biosystems). Oligonucleotides were removed from pooled PCR product library using the QIAquick^®^ PCR Purification Kit (Qiagen, Inc.). Single end sequencing of V2 hypervariable region was performed on Illumina MiSeq next generation sequencing platform using the v3 kit (a service provided by The Estonian Genome Center Core Facility).

### Bioinformatics

Reads with low quality (Q<30) and shorter than 225 bp (basepairs) were removed from the dataset. Operational taxonomic unit (OTU) was defined using the average neighbor-clustering algorithm of MOTHUR 1.34.4 [34] with 97% similarity threshold. Reference sequences were selected from the SILVA ribosomal RNA database release 102 [35]. The UCHIME algorithm was used to remove chimeric DNA sequences caused by PCR errors [36]. Taxonomic assignments were processed by the Ribosomal Database Project (RDP) naïve Bayesian Classifier [37] and BLAST [38] searched against a NCBI (National Center for Biotechnology Information) nucleotide database (release 206, http://www.ncbi.nlm.nih.gov/). Sequences matching eukaryotic DNA were discarded from the dataset. Statistical analyses were carried out with program R version 3.1.2 (http://www.r-project.org), ACE (Abundance-based Coverage Estimation) [39] and Chao1 [40] richness estimates were calculated using VEGAN package [41]. The same package was used to carry out the non-metric multidimensional scaling (NMDS) analyses. Week number was used as an independent variable for time. For data visualizations ggplot2 package was used [42]. All sequences are uploaded to the GenBank and are available under accession numbers KT855220–KT860044.

## Results

### Physicochemical factors

**Fig 2:**
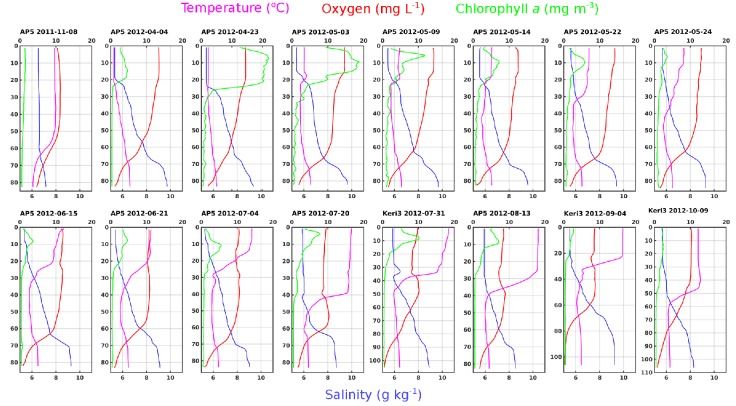
Water column profiles of stations AP5 and Keri3. Temperature (°C, magenta), oxygen (mg/L, red) and chlorophyll *a* (mg/m^3^, green) are on upper x-axis. Salinity (g/kg, blue) is on bottom x-axis. The sampling date is marked as following: YYYY-MM-DD.

The study covered the central region of the Gulf of Finland and was carried out on two transects (Fig 1). These transects provided both vertical and horizontal spatial coverage of the area. Dramatic shifts in environmental parameters took place over the sampling period (Fig 2). The halocline at 60–80 m depth is quasi-permanent in the gulf because of the freshwater inflow from rivers in the surface layer and water exchange with Baltic Proper in the near-bottom layer. Samples collected from 5 m horizon had an average salinity of 5.58 g kg^-1^(n = 63, SD = 0.25) and on the other hand, > 60 m layer had an average salinity of 8.70 g kg^-1^ (n = 70, SD = 0.68). The dissolved O_2_ concentration had negative co-variation with salinity and corresponding average values for O_2_ were 11.3 (SD = 2.2) and 1.8 mg L^-1^ (SD = 1.4, MIN = 0.0). The temperature remained quite stable in the near-bottom layer, with on average 4.6°C (SD = 0.45), but the surface layer temperatures varied in a large extent ranging from just under 1°C in the beginning of April and reaching up to 18°C in July. The thermocline became more pronounced in June. Three phytoplankton blooms typically occur in the Baltic Sea: spring, summer and autumn blooms; in decreasing order by biomass [43]. Consequently, the highest chlorophyll *a* concentrations in the surface layer were recorded during spring (almost up to 15 mg m^-3^), followed by summer and autumn values.

### Picoplankton cell counts

Highest cell count numbers were recorded at the surface layer (< 25m, n = 34; S1 Fig) reaching up to 3.6 x 10^6^ cells ml^-1^ (mean = 1.9 x 10^6^, SD = 0.7 x 10^6^), and as expected the cell abundance had significant correlation with temperature (r = 0.60, p < 0.001; Pearson). At the intermediate layer (25–65 m, n = 68) usually lowest cell counts were observed (mean = 3.93 x 10^5^ cells ml^-1^, SD = 2.6 x 10^5^, n = 37) and at the hypoxic/suboxic near-bottom layer (>65 m, n = 35) the picoplankton abundance increased compared to overlaying water, with on average 0.79 x 10^6^ cells ml^-1^ (SD = 4.12 x 10^5^). The double-stranded DNA staining technique used does not provide discrimination between *Bacteria* and *Archaea*, therefore, obtained results reflect overall prokaryotic picoplankton abundances.

### Observed and estimated bacterioplankton diversity

After quality-filtering and removal of chimeric and eukaryotic sequences, a total of 955,023 16S rRNA gene hypervariable region V2 reads were used in this analyses. These sequences were divided between 181 different samples, with on average 5,310 (SD = 3,070, MIN = 1,840, MAX = 23,720) sequences per sample. A total of 4,692 OTUs (97% similarity cut-off) were obtained, out of which 2,840 were singletons (single-read OTUs). Chao1 and ACE species richness estimates for each sample are listed in S2 Table. There were only small differences between the estimates. Chao1 species richness estimates were recorded highest at the near-bottom layer, with on average 394 OTUs (SD = 168, n = 61). At the intermediate layer the estimates were slightly lower, with on average 344 OTUs (SD = 125, n = 62); and at the surface layer the average estimated diversity was lowest (mean = 299, n = 58), however the variability was highest (SD = 329). The Chao1 species richness estimates were significantly higher at the near-bottom layer than at the surface and intermediate layers (t-test, p<0.1 in both cases). There was no statistical difference between the two upper layers (t-test, p = 0.33).

### Dynamics of BCC

**Fig 3:**
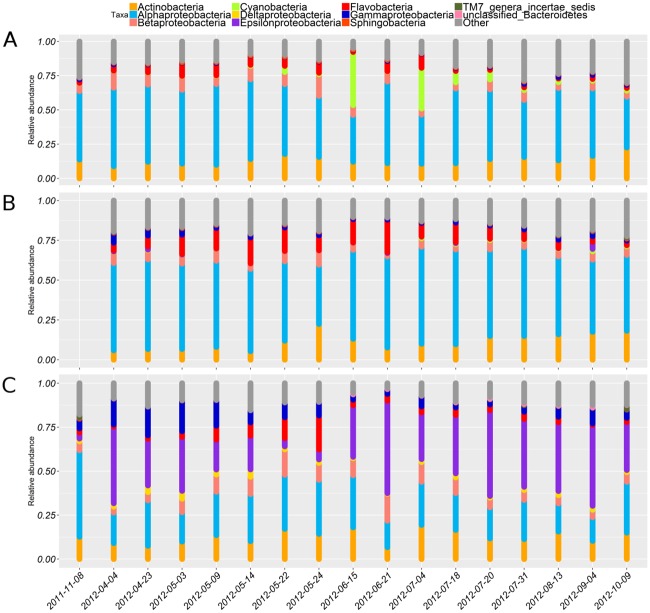
The relative abundance of dominant bacterial classes given in three depth ranges: 5–25 m (A), 25–65 m (B) and >65 m (C).

Eleven bacterial classes accounted for 86.4% of overall BCC and spatiotemporal patterns of these classes help to decrypt the dynamics of the community structure on a broader scale (Fig 3). *Alphaproteobacteria* contributed the largest fraction of the dataset, mainly occupying the upper oxygenated water (on average 51.1% of BCC above 65 m; Fig 3). Their dominance at the surface layer was only challenged by *Cyanobacteria* during summer bloom (reaching 53.7% of BCC; Fig 3). On the other hand, there were also bacterial classes that occurred mainly in hypoxic/suboxic near bottom layer, constituting of mostly *Epsilonproteobacteria*, but also of *Delta-*, *Gammaproteobacteria*, and one class level group that could be classified only on phylum level (unclassified *Bacteriodetes*). Below 65 m these groups contributed on average 31.7%, 19.4%, 6.9% and 0.6% of BCC; respectively. The relative abundance of *Gammaproteobacteria* peaked in April (21.7% of BCC; Fig 3).

*Actinobacteria*, *Betaproteobacteria*, and *Flavobacteria* were found throughout the water column (Fig 3). These bacterial classes contributed on average 12.9%, 6.6% and 5.8 of BCC; respectively. However, their occurrence revealed distinct spatiotemporal dynamics. *Betaproteobacteria* and *Flavobacteria* contributed to a larger fraction in spring, increasing in relative abundance in the intermediate as well hypoxic near-bottom layer (Fig 3). The fraction of *Actinobacteria* became more predominant in late summer and peaked in autumn (up to 36.4% of BCC), although its relative abundance also peaked at the end of May at intermediate and near-bottom layers (Fig 3). TM7 genera *incertae sedis* appeared only in autumn samples, with a maximum relative abundance of 3.7% (Fig 3).

**Fig 4:**
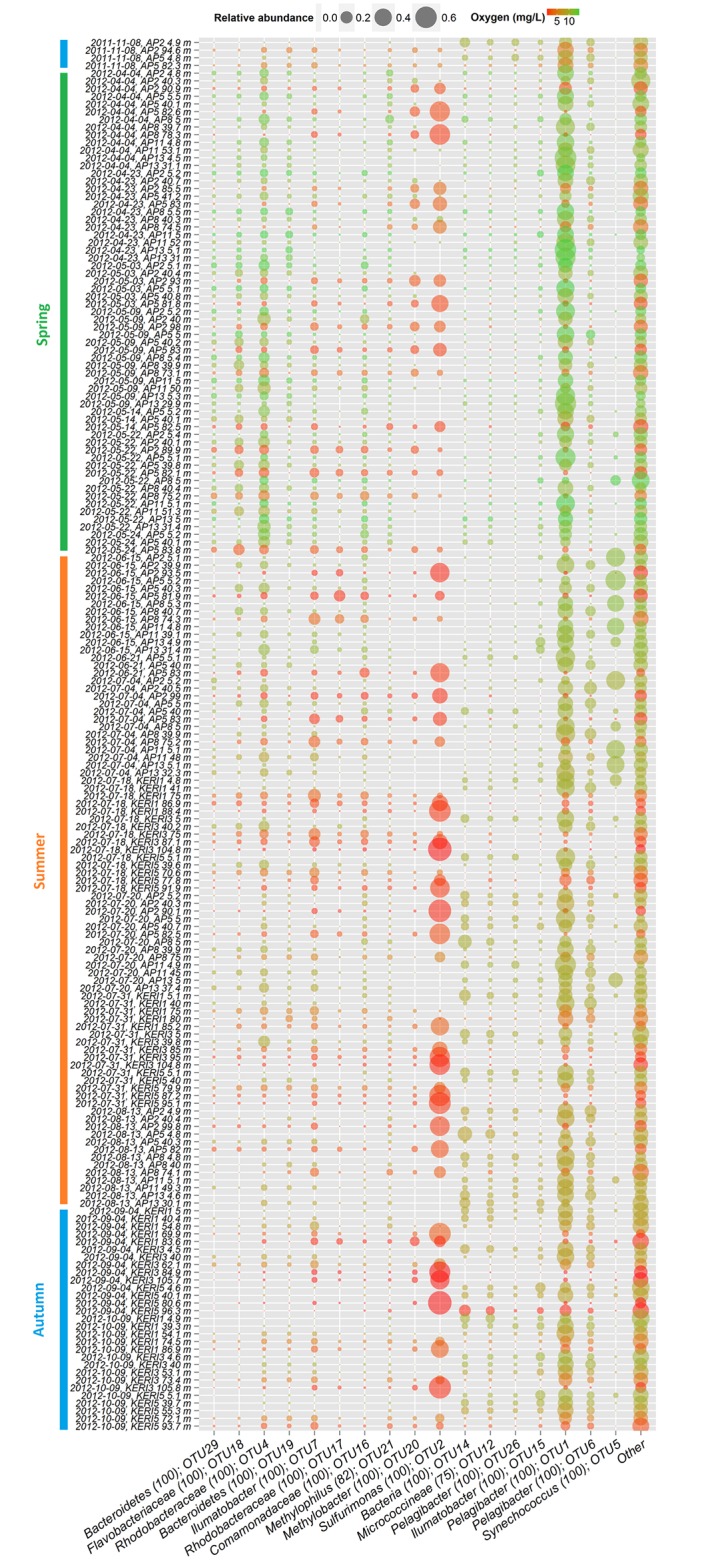
Occurrence patterns of relatively abundant OTUs (top 17). OTUs are ordered by their co-localization.

At the species level, the bacterioplankton community structure was much more complex, despite the fact that sequence distribution between OTUs was uneven. Sorted by their relative abundance, the top 17 OTUs accounted for more than 1% of the total dataset each, adding up to 76% of the total dataset and are henceforth referred to as ‘relatively abundant OTUs’ (Table 1, Fig 4). The following 56 OTUs in relative abundance were in the range of 0.1–1% (totalling up 16%; S2 Fig) and can be considered as ‘common’ [24]. Last 4619 OTUs contributed less than 0.1% (i.e. ‘relatively rare’). Moreover, when abundance cut-off for relatively rare taxa was set to 0.01%, then still 4440 OTUs met the criteria, leaving 179 OTUs in the range 0.01–0.1%. However, considering occurrence in relation to the whole dataset discriminates against opportunistic ‘pulse’ populations that can be relatively abundant in limited time-space and therefore ecologically relevant. There were 101 OTUs that contributed a minimum of 1% of BCC in at least one of the samples. Lowering the threshold to 0.1%, this number quadrupled and resulted in 413 OTUs.

**Table 1:** Relatively abundant OTUs, their taxonomic affiliation according to the RDP classifier, their closest neighbours in the GenBank database with isolation source.

OTU	Classification (classification confidence—%)	Genbank accession	Similarity score	Isolation source
OTU1	‘*Candidatus* Pelagibacter’ (100)	EU801724	0.987	Chesapeake Bay, MD
OTU2	*Sulfurimonas* (100)	KC492833	0.987	Baltic Sea redoxcline, 119 m depth
OTU4	*Rhodobacteraceae* (100)	JX015552	0.987	marine bulk water (the North Sea)
OTU5	GpIIa (100)	AY151238	0.991	isolate
OTU6	‘*Candidatus* Pelagibacter’ (100)	EU799240	1.000	Newport Harbour, RI
OTU7	*Ilumatobacter* (100)	HM446118	0.987	West Thumb 98 (Yellowstone Park)
OTU12	*Micrococcineae* (75)	DQ316352	0.992	freshwater
OTU14	*Bacteria* (100)	EU801283	0.992	Chesapeake Bay, MD
OTU15	*Ilumatobacter* (100)	EU800762	0.996	Delaware Bay, NJ
OTU16	*Comamonadaceae* (100)	FR685984	0.995	marine biome, fjord, coastal water
OTU17	*Rhodobacteraceae* (100)	HQ153846	0.983	shallow hydrothermal vent (depth of 189.1 m)
OTU18	*Flavobacteriaceae* (100)	AM279175	0.983	marine water (*Gymnodimium catenatum*)
OTU19	*Bacteroidetes* (100)	JX015752	0.995	marine bulk water (the North Sea)
OTU20	*Methylobacter* (100)	AF152597	0.991	isolate
OTU21	*Methylophilus* (82)	AJ400352	0.987	North Sea
OTU26	‘*Candidatus* Pelagibacter’ (100)	EU800094	0.996	"Delaware Bay, NJ
OTU29	*Bacteroidetes* (100)	GQ259245	0.942	surface water (the Arctic ocean)

Variability of BCC in relation to environmental conditions was analysed using detrended correspondence analysis (DCA) with added linear fitting of environmental parameters onto the ordination (S3 Fig). The DCA was chosen for all of samples and OTUs because it helps to overcome the ‘arch effect’ that accompanies correspondence analyses. The relevance of salinity, depth, and oxygen were statistically highest, yielding r^2^ values of 0.773, 0.742 and 0.630 (P<0.001 in each case), respectively. These three parameters were followed by temperature (r^2^ = 0.289, P<0.001).

**Fig 5:**
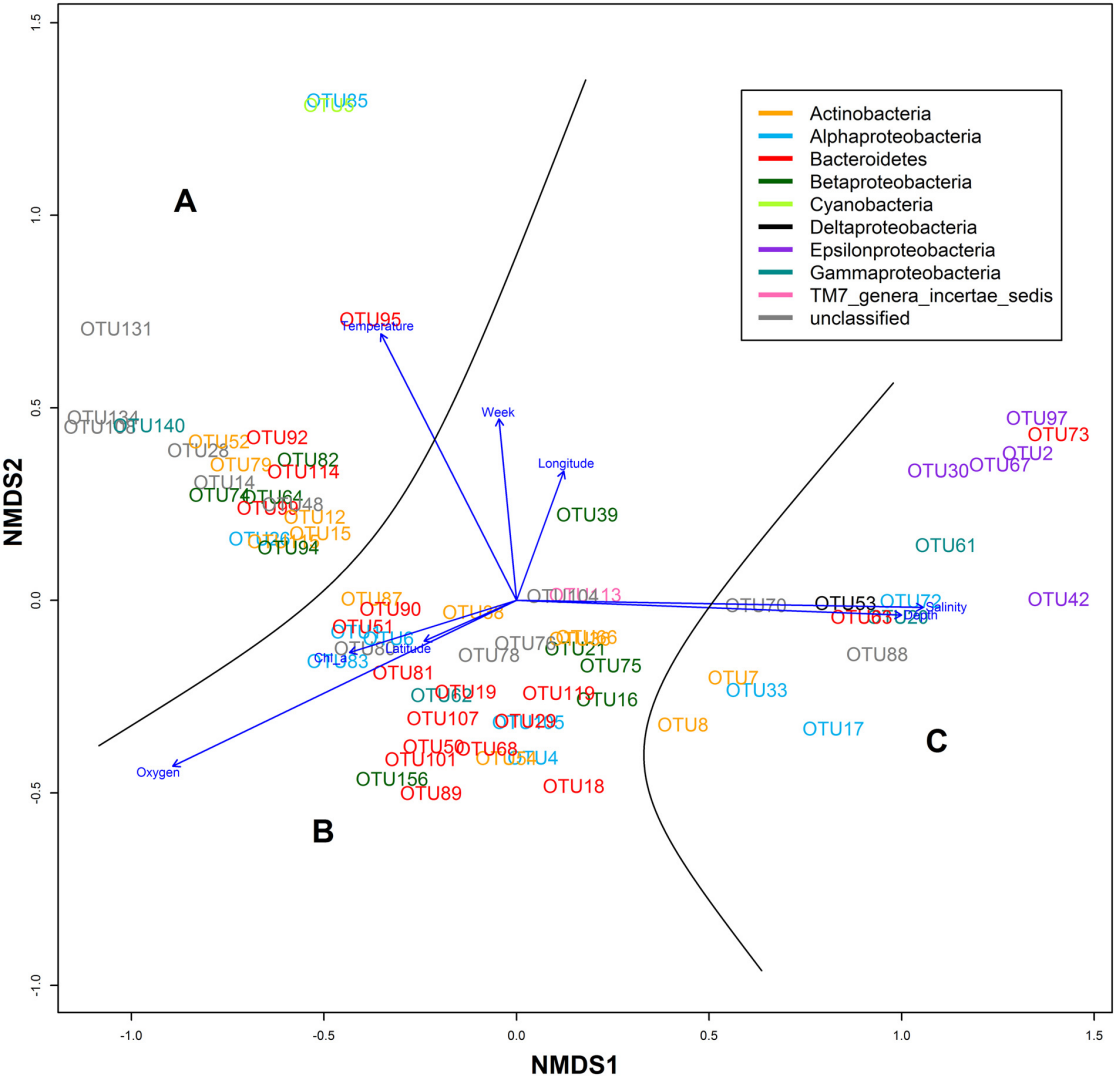
NMDS plot of abundant and common OTUs fitted with environmental parameters (stress score: 0.125). The added black lines divide OTUs into three major groups, which are marked with letters for discussion.

Non-metric multidimensional scaling (NMDS) analysis of relatively abundant and common OTUs was carried out (based on Bray-Curtis dissimilarity matrix) and supplemented with the linear fitting of environmental parameters onto the ordination (Fig 5). As a result, three general groups can be distinguished: OTUs that were mainly found in surface layer in summer and autumn (group A); spring phytoplankton bloom-associated OTUs, including populations that became more prevalent in deeper layers after the bloom had ended (group B); and OTUs that were mostly found in hypoxic waters (group C in Fig 5). Therefore, seasonal succession (with important impact of changing temperature); and a covarying trio of salinity, pressure and oxygen could be established as main factors influencing the occurrence of these OTUs.

Most dominant populations in the ‘hypoxic group’ were members of *Epsilonproteobacteria*: OTU2, OTU30, OTU67, OTU97 (classified as *Sulfurimonas*) and OTU42 (classified as *Arcobacter*). OTU2 contributed up to 71% of BCC and had 99% sequence similarity to the representative of *Sulfurimonas* GD1/GD17 subgroup, which has been shown to be key chemolithoautotrophic taxa in the Gotland Deep [44, 45].

The major bulk of *Alphaproteobacteria* was contributed by OTUs classified as “*Candidatus* Pelagibacter” (OTU1, OTU6, OTU26, and OTU83). OTU1 and OTU6 remained transiently abundant with minor variations in time (Fig 4, S2 Fig). OTU26 was more prominent at surface layer towards the autumn (Fig 4) and clustered together with relatively abundant actinobacterial OTUs: OTU12 (*Micrococcineae*), OTU15 (*Ilumatobacter*) and OTU14 (closest database affiliation to *Micrococcineae*; Table 1). Some of these OTUs exhibited similar presence in samples collected autumn in 2011 and 2012 (Fig 4, S2 Fig).

**Fig 6:**
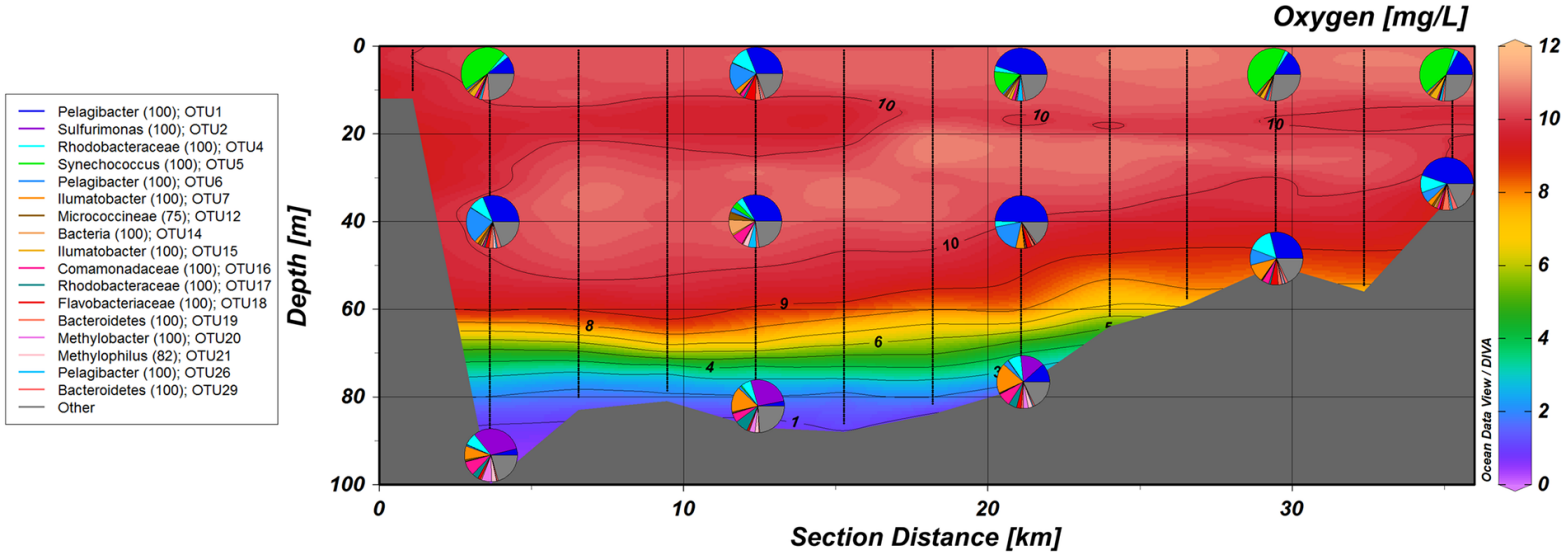
Cross-section of AP-transect on 2012-07-04 supplemented with relative abundances of top 17 OTUs.

Unicellular cyanobacterium *Synechococcus* (OTU5) and co-occurring OTU85 (*Rhodobacteraceae*) formed a separate cluster (Fig 5), and a significant and strong relationship between OTUs and temperature suggests that temperature had a strong impact on their occurrence. OTU95 (*Flavobacteriaceae*) and OTU39 (classified as *Alcaligenaceae*) appeared during the peak of *Synechococcus* relative abundance, only in deeper layers (S2 Fig). During summer bloom *Synechococcus* displayed a patchy distribution at the surface layer above the thermocline as its relative abundance varied along AP-transect; members of “*Candidatus* Pelagibacter” remained dominant in the intermediate layer, and representatives of *Sulfurimonas* contributed the largest fraction at the hypoxic near-bottom layer (Fig 6).

**Fig 7:**
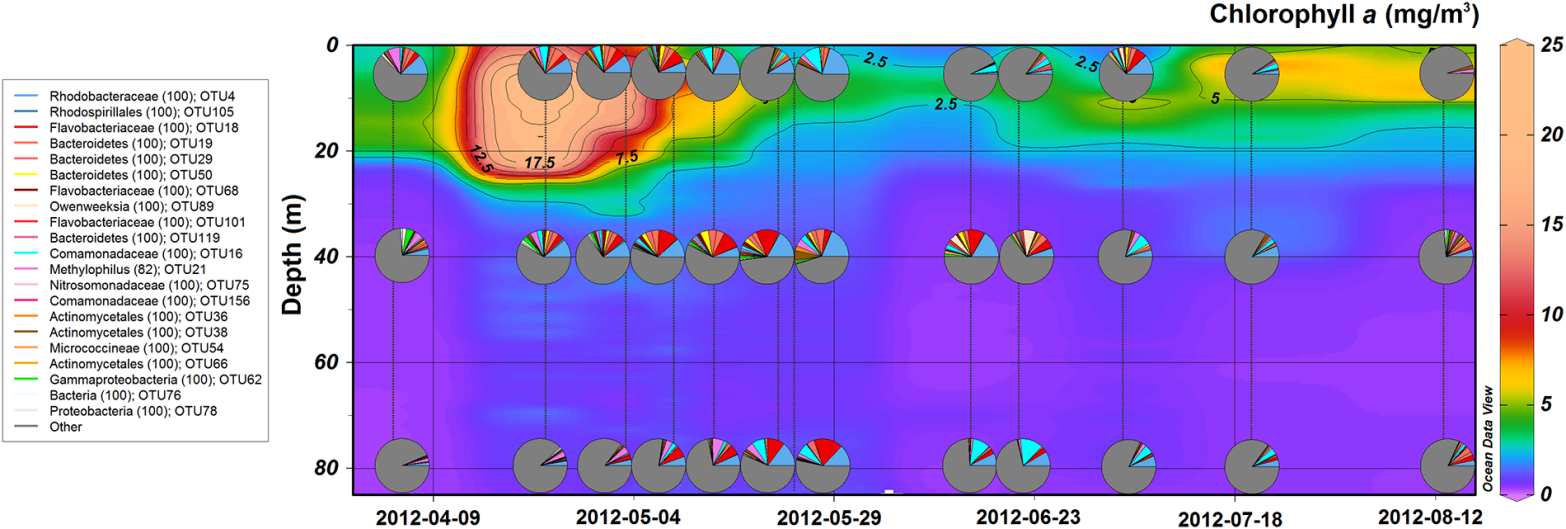
Time series of Chlorophyll *a* at station AP5. Pie charts indicate the relative abundance of OTUs that were associated with the spring phytoplankton bloom. Note that the second surface community (2012-04-23, 5 m depth) was recorded at station AP2 not AP5.

Relatively abundant and common OTUs associated with the spring phytoplankton bloom could also be distinguished as a separate co-localizing cluster in the middle bottom of NMDS plot (Fig 5). This positioning is explained by negative correlations with time and their spread throughout the water column, which resulted in weak correlations with dissolved oxygen concentrations. These OTUs contributed a large fraction of BCC above the halocline during the spring bloom and remained abundant at intermediate layer till mid-summer (Fig 7). The most relevant bacterial populations by their relative abundance were classified as *Rhodobacteraceae* (OTU4), *Comamonadaceae* (OTU16) and *Bacteroidetes* (OTU19 and OTU29). The fraction of these OTUs also increased in the near-bottom hypoxic layer as the spring bloom progressed (Fig 7). Interestingly, betaproteobacterial OTU16 (*Comamonadaceae*) reached its maximum abundance in hypoxic conditions in the beginning of summer (Fig 7). During that period also OTU7 (*Ilumatobacter*) and OTU17 (*Rhodobacteraceae*) became more predominant in the hypoxic layer (reaching 10.3% and 17.8% of BCC, respectively), but the opposite trend was displayed by OTU2 (Fig 4). OTU21 (*Methylophilus*) was present at deeper layers throughout the sampling period; however, it contributed a larger fraction during the spring bloom and had its maximum abundance occurred at 5 m depth at the beginning of April (Fig 7).

## Discussion

High-throughput marker gene sequencing has opened a new gateway to investigate aquatic microbial communities. The present study explores the abundances of picoplankton and BCC of the Gulf of Finland in three dimensions: horizontally from south to north, vertically from the surface to near-bottom layer, and temporally from spring to autumn. Similar investigations are quite rare as most temporal studies lack spatial dimension and *vice versa*. The focus of this study is on spatiotemporal patterns and phylogenetic relationships of OTUs that are relatively abundant or common. However, our results and previous investigations of BCC of the Baltic Sea [12, 15–17, 22, 24] also reflect the presence of the ‘rare biosphere’, which is represented by the long tail pattern found in rank-abundance curves of bacterial species [46–48]. That long tail is composed mostly of dormant cells and can produce a pulse of ecosystem activity by resuscitation [49], acting as a reservoir of genetic and functional diversity [50]. The presence of the ‘rare biosphere’ is also reflected from our present results, as well as from previous investigations of BCC of the Baltic Sea using next-generation sequencing platforms [12, 15–17, 22, 24]. However, the focus of this study is on spatiotemporal patterns and phylogenetic relationships of OTUs that are relatively abundant or common. The switch point between the ‘tail’ and the common or abundant bacterial populations is, of course, subjective, and 0.1% of the total dataset was selected for this analysis [51].

### Spatiotemporal niche partitioning of the BCC

The approach used, massively parallel sequencing of the amplified 16S rRNA gene fragment libraries, enabled culture-independent and detailed view in the dynamics of bacterioplankton community. Spatiotemporal surveys of BCC in ecosystems with sharp environmental gradients (like river mouths, hypoxic estuaries or coastal regions) have demonstrated that spatial variation overpowers seasonal succession [6, 7, 16]. Our results reflect the same trend, but also demonstrate in fine detail the seasonal succession throughout the water column. There were 17 relatively abundant OTUs. However four dominant OTUs (OTU1—“*Candidatus* Pelagibacter”, OTU2—*Sulfurimonas*, OTU4 –*Rhodobacteraceae* and OTU5—*Synechococcus*) could be considered as core populations of bacterioplankton in the Gulf of Finland.

There is no sill between the Gulf of Finland and Baltic Proper, hence the deep inflow to the gulf takes place just below the co-occurring oxy- and halocline [28]. Consequently, taxa characteristic of the sulfidic zone of the central Baltic Sea are dispersed into the Gulf of Finland [15–17]. Our results of agree well with findings from these studies. Nitrate-reducing and sulphide-oxidizing *Sulfurimonas gotlandica* (98.7% similarity to OTU2) is capable of motility and chemotaxis; hence, it is typically more abundant near the oxycline [52, 53]. In contrast, sulfate-reducing *Deltaproteobacteria* are more prominent in deeper anoxic layers (below 140 m) [54]. As a consequence, *Deltaproteobacteria* contribute small and *Sulfurimonas* make up large fraction of the bacterial community in the near-bottom layer of the Gulf of Finland (Fig 3). The sulphur reducers in the gulf are mainly found in the sediments [55].

Alphaproteobacterial SAR11 clade (candidate order “*Pelagibacterales*”) and also Roseobacter group constitute high proportions of the marine bacterioplankton globally and in the geographically close North Sea [55–58]. Both of these groups were made up OTUs that can be considered core populations for the area. The Baltic Sea is known to harbour several phylogenetic lineages of the “*Pelagibacterales*” that exhibit niche partitioning along the salinity gradient [12, 54]. Results from the present and previous studies indicate that this group is a transiently abundant heterotrophic group in the surface layer (S2 Fig, Fig 6) [16, 17]. Members of SAR11 clade and order *Rhodobacterales* are mostly involved in the transport and metabolism of low-molecular-weight organic compounds [58–60]. The latter includes degradation of phytoplankton-produced dimethylsulfoniopropionate (DMSP) to dimethylsulfide (DMS) [61]. It is highly probable that these groups facilitate these processes also in the Gulf of Finland, especially given that OTU4 had its highest occurrence during the spring phytoplankton bloom.

The coordinated and cooperative activities of bloom-associated ‘specialists’ are critical in the biogeochemical cycles of the ecosystem for determining the fate of the organic biomass. Various members of *Actinobacteria*, *Betaproteobacteria*, *Bacteroidetes* and *Verrucomicrobia* have been demonstrated to be responsible for the degradation of high-molecular-weight organic compounds in the Baltic Sea [21, 62, 63] and in the North Sea [58, 64]. In general, temporal dynamics of these groups at the surface layer are in line with previous investigations [22, 24]; for example, representatives of *Bacteroidetes* were more abundant during spring and early summer, and members of *Actinobacteria* were prevalent in late summer and autumn (Fig 4). However, to the best of our knowledge, the present study is the first that provides simultaneous insight into dynamics of BCC below surface layer in this region (Fig 7, S2 Fig).

Annually recurring spring phytoplankton blooms in the Gulf of Finland consist mainly of diatoms and dinoflagellates [65]. During spring bloom, a large fraction of annual primary production is carried out [43] and therefore these bloom-associated bacterial populations hold great ecological significance. Our results demonstrate shifts in BCC during the bloom (Fig 7) and likely sedimentation of some OTUs to the deeper layers. The fraction of bloom-associated populations steadily increased at near-bottom hypoxic layer probably due to attachment to decaying phytoplankton cells or other settling particles (Fig 7). It is important to keep in mind that bacteria attached to particles or aggregates larger than 5.0 μm were excluded from the dataset due to prefiltration. However, certain bacterial lineages are known to switch between free-living and particle-attached lifestyle and also some of the particle-associated groups can get detached during the filtration process. These possibilities have to be considered, especially because there is a clear influx of populations into the deeper layers that in previous data points were prevalent in the surface layer.

At the beginning of the spring bloom, OTU4 (Roseobacter group) betaproteobacterial OTU21 (*Methylophilus*) and three members of *Bacteroidetes* (OTU18, OTU19, and OTU29) constituted a considerable fraction of BCC. The presence of OTU21 shifted rapidly from the surface to deeper layers (Fig 7). Representatives of the *Methylophilaceae* family (*Betaproteobacteria*) have recently been identified as dominant DMS-degrading populations in Tocil Lake sediment and soil collected from *Brassica oleraceae* field [66]. This finding suggests that two relatively abundant and common OTUs that were classified as *Methylophilus* could also be metabolizing DMS, especially considering that OTU21 had its closest affiliation to sequence isolated from samples from the North Sea after an algal bloom (Fig 7).

The spatiotemporal patterns of bloom-associated bacterial populations provide valuable insight into mechanisms of community assembly in relation to the phytoplankton community and the fate of organic matter produced by phytoplankton. One important aspect of the current study is to shed light on phytoplankton bloom related shifts in BCC that take place in deeper hypoxic layers. To start off, the relative abundance of representatives of *Bacteroidetes* decreased at the surface layer as the bloom progressed, but at 40 m depth, some OTUs remained present until the beginning of summer (Fig 7). However, by the end of the spring bloom OTU18 (*Flavobacteraceae*) contributed a large fraction of BCC at the near-bottom hypoxic layer at the end of May (Fig 7). This could occur due detachment from lysing phytoplankton cells. However, the occurrence of unclassified *Flavobacteraceae* in oxygen depleted water was also observed during the spring phytoplankton bloom in Byfjord; in the following year, when oxygen conditions improved, the abundance of that group was drastically reduced [7]. This gives an indication that degradation of organic matter in hypoxic conditions provides a specific niche, especially considering that some of the members of *Bacteroidetes* were mostly found in the oxygen depleted near-bottom layer (OTU63 and OTU73; Fig 5).

Similarly, betaproteobacterial OTU16 (*Comamonadaceae*) appeared at surface layer in the end of the spring bloom and peaked in abundance after sedimentation at hypoxic layer in the beginning of summer. Members of *Comamonadaceae* have shown to be involved in denitrification and to be active in the hypoxic zone [67, 68]. In addition, OTU7 (*Ilumatobacter*) became more abundant in the hypoxic layer during the spring bloom; the same group was associated with diatom degradation in the near-bottom layer of Lake Baikal [69].

The spatiotemporal patterns of these populations provide valuable insight into mechanisms of community assembly. The presence of certain bloom-associated populations is affected not only by phytoplankton community but also by the presence of a hypoxic zone. The fact that the abundance of some of the OTUs increased in the hypoxic zone suggests that they are probably capable of switching the terminal electron acceptor, and therefore the oxygen depleted zone provides a particular niche for them. These results point toward combined effects of substrate- and redox-driven niche partitioning on the BCC.

In addition to substrates provided by phytoplankton, there are compounds produced by bacteria in the sediments, like methane [70], which as powerful greenhouse gas bears global significance. The abundance and activity of methane-oxidizing bacteria in the water column are crucial regulators of methane emission to the atmosphere. Our results demonstrate a vertical distribution of different potentially methanotrophic bacteria. The most dominant of which were classified as *Methylobacter*, like OTU20, which was mainly found in the hypoxic near-bottom layer, where the methane concentrations are potentially highest [70, 71]. Also, OTU21 and OTU94 were classified as *Methylophilus*, a non-methane-oxidizing bacterial group which usually utilizes other C1 substrates [72]. However, the representatives of *Methylobacter* and *Methylophilus* have been demonstrated to cooperate in methane utilization [73, 74] and experiments with such methane-consuming communities suggested that, in low oxygen concentrations, members of *Methylobacter* dominate over representatives of *Methylophilus* and *vice versa* [74]. Our *in situ* results confirm these previous findings. Overall, our results reflect the importance of C1 substrate utilizing bacterial populations in the gulf as they contribute a significant fraction of BCC throughout the water column (Fig 4).

### Emerging pattern of cosmopolitan specialists

It is well established that salinity has a major impact on bacterioplankton community assemblage, gene expression and metabolic activity in aquatic ecosystems, including the Baltic Sea [12, 20, 75]. Over the past decade, there has been accumulating evidence that certain phylogenetic lineages (for example members of SAR11, *Rhodobacterales*, *Methylophilales*, *Synechococcus*, SUP05) contribute a significant fraction of BCC in geographically distant, but hydrographically close (brackish and periodically/partially hypoxic) estuaries [7, 16, 76–78]. Moreover, recent genome level comparisons using metagenome-assembled genomes have demonstrated that the Baltic Sea and the Chesapeake Bay harbour not only phylogenetically close members, but exactly the same species [59]. The similarity of communities in distant locations fits the oft-repeated ‘everything is everywhere, but the environment selects’ concept in ecology [79, 80]. This concept agrees well with the species-sorting paradigm of metacommunity theory [81], by which the geological barriers are rendered irrelevant and local environmental conditions play a central role in the community assembly process. From our previous studies, we have concluded that these communities can be considered as a metacommunity, and now this statement is backed with more evidence [16].

Hugerth *et al*. [59] put the selective emphasis on the salinity range, coining it a ‘brackish microbiome’, but their analyses included reads obtained only from the sea surface communities. However, the similarities in BCC were observed with estuaries also suffering from oxygen depletion. The results of the present study clearly demonstrate the structuring effect of dissolved oxygen, because some bacterial populations achieve an advantage in the hypoxic zone through very likely using alternative electron acceptors (e.g. denitrification). The notable presence of usually oxic surface bacterial populations also in the hypoxic zones has raised questions of their anoxia-tolerance and capability to use alternative electron acceptors [16, 82]. Functional capability to use alternative electron acceptors gives a distinct advantage to bacteria in such oxygen depleted ecosystems and can provide specific niches as demonstrated by bloom-associated OTUs (Fig 7).

Thereby, rather than putting salinity or redox conditions in the central role in bacterioplankton community assemblage in the Baltic Sea, it should be looked as a combination of the two. Hence, these communities can be considered as metacommunity driven by gradients of redox conditions and salinity. An analogy would be a symphony orchestra on a world tour: it plays the same melodies (ecosystem services), and although some musicians (bacterial populations) may vary between concerts, they play the same instrument (functional niche). In the case of the Baltic Sea, one of these ‘unique musicians’ is *Sulfurimonas gotlandica*, as sulfur-oxidation in most of these other communities is carried out by SUP05 clade members [7, 76–78, 82].

## Conclusions

The present study provided a detailed insight into spatiotemporal patterns of BCC in the Gulf of Finland, a stratified estuary suffering from oxygen depletion in the near bottom layer. Thousands of OTUs were identified in the framework of this study, but most of them belong to the rare biosphere, which can be accessed by ‘deep’ sequencing. Only around one hundred OTUs could be considered relatively common or abundant. Oxygen availability explained most of the variability of BCC, however, seasonal dynamics were observed both in oxygenated and hypoxic layers. Especially intriguing were the spatiotemporal occurrence patterns of heterotrophs responsible for degradation of the spring phytoplankton bloom-derived organic matter, as a combination of substrate- and redox-driven niche partitioning could be observed. These spatiotemporal niche specializations among bacterioplankton communities are crucial for predicting ecosystem functioning and understanding multilevel effect of oxygen depletion to the Baltic Sea, and in addition, help explain biogeography of certain cosmopolitan species.

## Supporting Information

S1 Fig Picoplankton total cell count numbers supplemented with oxygen concentration (mg/L). (TIF)

S2 Fig Occurrence patterns of abundant and common OTUs (top 73). OTUs are ordered by their co-localization.
(TIF)

S3 Fig Detrended correspondence analysis of the bacterioplankton community composition fitted with environmental parameters. Red crosses represent individual OTUs (n = 4692) and circles represent different samples (n = 181).
(TIF)

S1 Table Sample collection metadata and physicochemical background data. (DOCX)

S2 Table Species richness estimates. (DOCX)
