# Supplementary figures and images for: Near-Bottom Hypoxia Impacts Dynamics of Bacterioplankton Assemblage throughout Water Column of the Gulf of Finland (Baltic Sea)
Source: PLoS One. 2016 May 23;11(5):e0156147. doi: 10.1371/journal.pone.0156147 (PMC4877108; doi:10.1371/journal.pone.0156147)

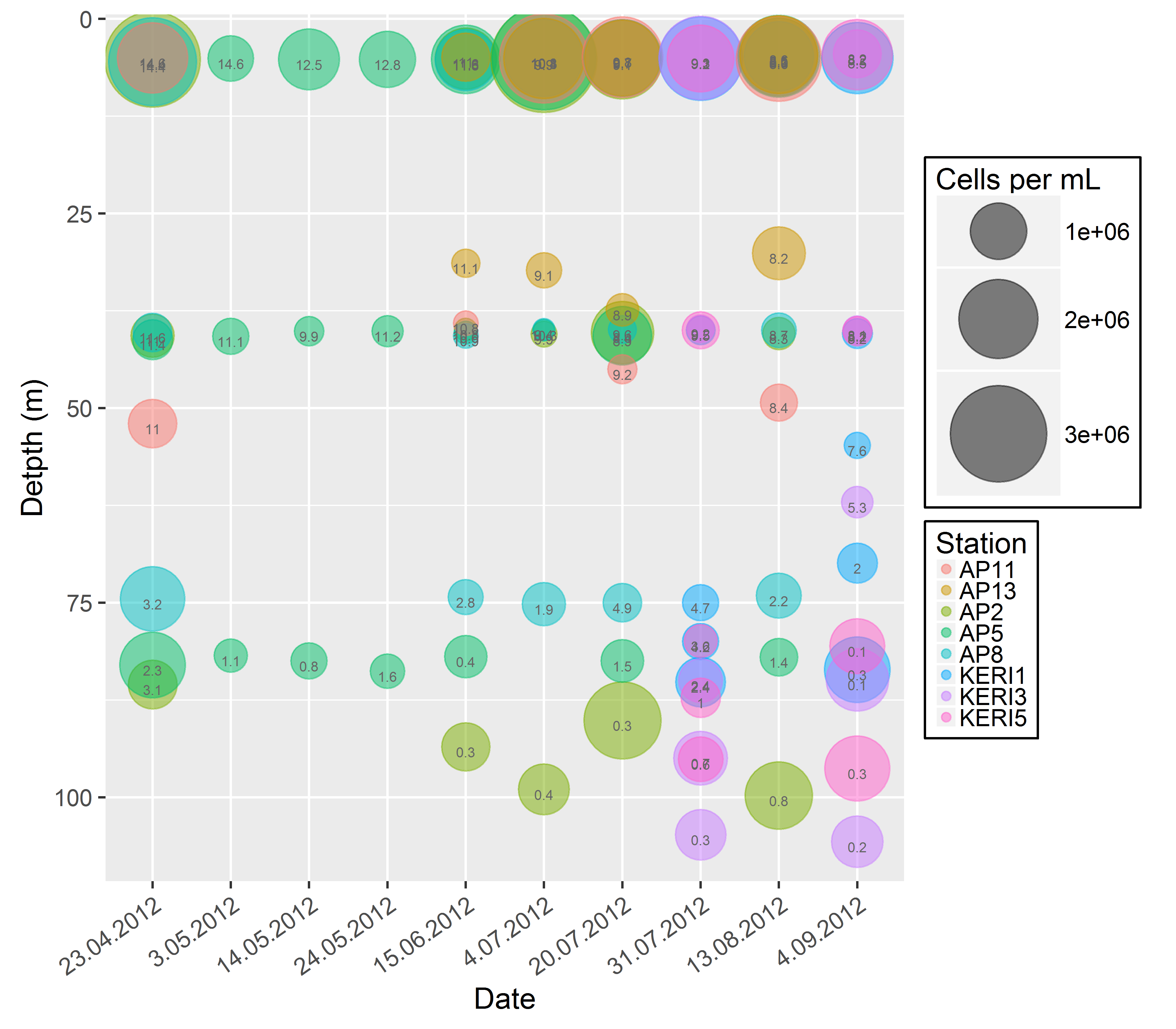

Supplement: S1 Fig — (TIF) [file pone.0156147.s001.tif]

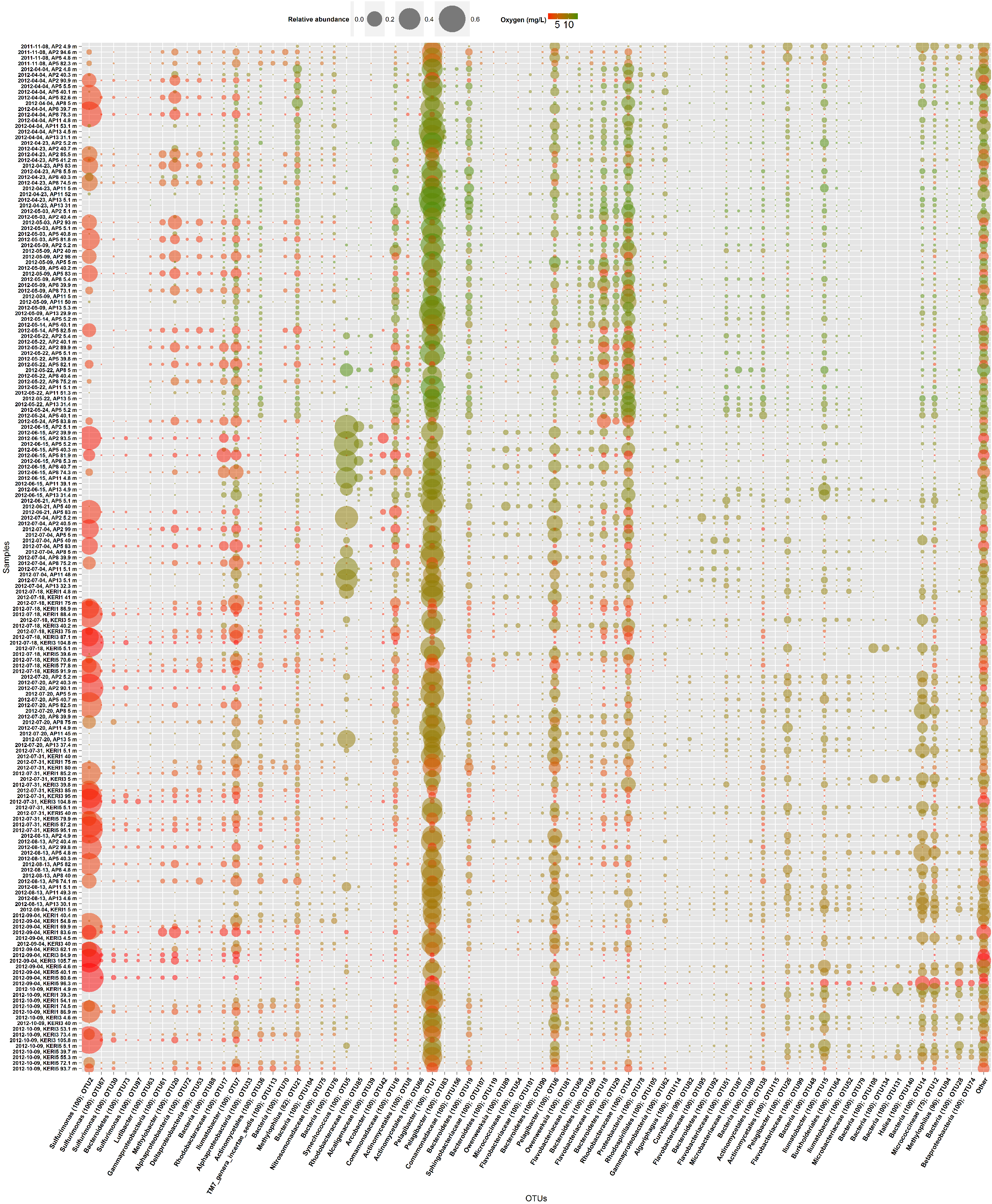

Supplement: S2 Fig — OTUs are ordered by their co-localization. (TIF) [file pone.0156147.s002.tif]

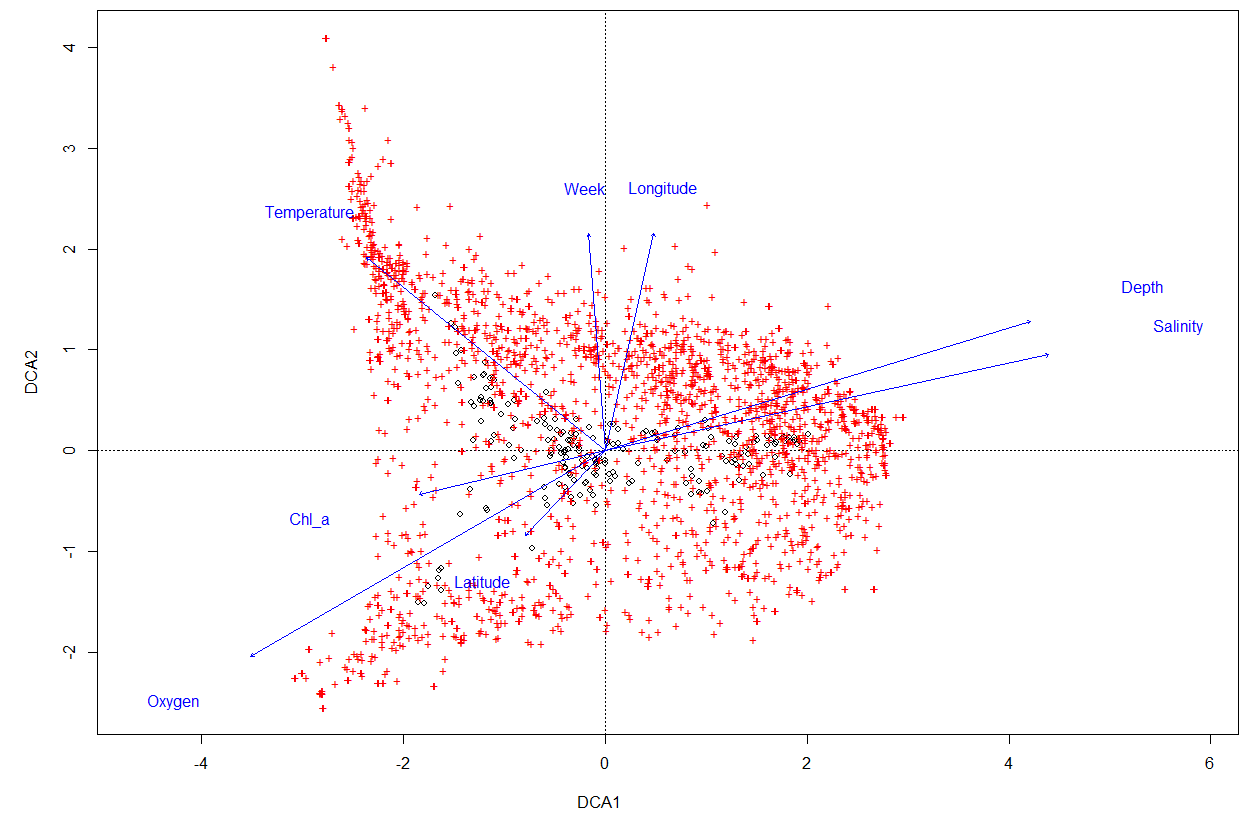

Supplement: S3 Fig — Red crosses represent individual OTUs (n = 4692) and circles represent different samples (n = 181). (TIF) [file pone.0156147.s003.tif]
