# Supplementary material for: Near-Bottom Hypoxia Impacts Dynamics of Bacterioplankton Assemblage throughout Water Column of the Gulf of Finland (Baltic Sea)
Source: PLoS One. 2016 May 23;11(5):e0156147. doi: 10.1371/journal.pone.0156147 (PMC4877108; doi:10.1371/journal.pone.0156147)
Supplement: S1 Table — (DOCX) [file pone.0156147.s004.docx]

|  | | | | | | | | | |
| --- | --- | --- | --- | --- | --- | --- | --- | --- | --- |
| **Date** | **Week** | **Station** | **Longitude** | **Latitude** | **Depth (m)** | **Tempe-rature (ͦC)** | **Salinity (g kg^-1^)** | **Chlorophyll *a* (mg m^-3^)** | **Oxygen (mg L^-1^)** |
| 8.11.2011 | 45 | AP2 | 24.69253 | 59.61823 | 4.9 | 10.11 | 6.58 | 0.44 | 10 |
| 8.11.2011 | 45 | AP2 | 24.69253 | 59.61823 | 94.6 | 3.53 | 7.36 | 0.2 | 3.6 |
| 8.11.2011 | 45 | AP5 | 24.62698 | 59.68858 | 4.8 | 9.63 | 6.53 | 0.54 | 10 |
| 8.11.2011 | 45 | AP5 | 24.62698 | 59.68858 | 82.3 | 3.64 | 7.17 | 0.2 | 4.5 |
| 4.04.2012 | 14 | AP2 | 24.69253 | 59.61823 | 4.8 | 0.68 | 5.25 | 2.51 | 13.2 |
| 4.04.2012 | 14 | AP2 | 24.69253 | 59.61823 | 40.3 | 3.01 | 6.91 | 0.15 | 10.5 |
| 4.04.2012 | 14 | AP2 | 24.69253 | 59.61823 | 90.9 | 5.35 | 9.62 | 0.14 | 1.3 |
| 4.04.2012 | 14 | AP5 | 24.62698 | 59.68858 | 5.5 | 0.89 | 5.34 | 1.19 | 13.4 |
| 4.04.2012 | 14 | AP5 | 24.62698 | 59.68858 | 40.1 | 2.47 | 6.77 | 0.16 | 11.3 |
| 4.04.2012 | 14 | AP5 | 24.62698 | 59.68858 | 82.6 | 5.37 | 9.74 | 0.14 | 1.2 |
| 4.04.2012 | 14 | AP8 | 24.56268 | 59.7582 | 5 | 0.93 | 5.4 | 2.26 | 13.3 |
| 4.04.2012 | 14 | AP8 | 24.56268 | 59.7582 | 39.7 | 2.34 | 6.78 | 0.15 | 11.3 |
| 4.04.2012 | 14 | AP8 | 24.56268 | 59.7582 | 78.3 | 5.41 | 9.84 | 0.15 | 0.7 |
| 4.04.2012 | 14 | AP11 | 24.50003 | 59.8278 | 4.8 | 1.38 | 5.48 | 2.18 | 13.2 |
| 4.04.2012 | 14 | AP11 | 24.50003 | 59.8278 | 53.1 | 2.96 | 6.66 | 0.2 | 10.5 |
| 4.04.2012 | 14 | AP13 | 24.45807 | 59.8758 | 4.5 | 1.65 | 5.58 | 1.79 | 12.9 |
| 4.04.2012 | 14 | AP13 | 24.45807 | 59.8758 | 31.1 | 2.26 | 6.07 | 0.41 | 11.5 |
| 23.04.2012 | 17 | AP2 | 24.69253 | 59.61823 | 5.2 | 2.7 | 5.45 | 9.96 | 14.4 |
| 23.04.2012 | 17 | AP2 | 24.69253 | 59.61823 | 40.7 | 2.72 | 7.04 | 0.29 | 11 |
| 23.04.2012 | 17 | AP2 | 24.69253 | 59.61823 | 85.5 | 4.9 | 9.11 | 0.16 | 3.1 |
| 23.04.2012 | 17 | AP5 | 24.62698 | 59.68858 | 41.2 | 2.48 | 6.89 | 0.52 | 11.4 |
| 23.04.2012 | 17 | AP5 | 24.62698 | 59.68858 | 83 | 5.08 | 9.36 | 0.16 | 2.3 |
| 23.04.2012 | 17 | AP8 | 24.56268 | 59.7582 | 5.5 | 2.38 | 5.42 | 10.18 | 14.4 |
| 23.04.2012 | 17 | AP8 | 24.56268 | 59.7582 | 40.3 | 2.09 | 6.73 | 0.3 | 11.6 |
| 23.04.2012 | 17 | AP8 | 24.56268 | 59.7582 | 74.5 | 4.91 | 9.16 | 0.24 | 3.2 |
| 23.04.2012 | 17 | AP11 | 24.50003 | 59.8278 | 5 | 2.54 | 5.47 | 11.3 | 14.6 |
| 23.04.2012 | 17 | AP11 | 24.50003 | 59.8278 | 52 | 2.64 | 6.89 | 0.53 | 11 |
| 23.04.2012 | 17 | AP13 | 24.45807 | 59.8758 | 5.1 | 2.45 | 5.46 | 7.19 | 14.5 |
| 23.04.2012 | 17 | AP13 | 24.45807 | 59.8758 | 31 | 2.48 | 5.65 | 4.2 | 13.4 |
| 3.05.2012 | 18 | AP2 | 24.69253 | 59.61823 | 5.1 | 4.02 | 5.5 | 9.4 | 14.3 |
| 3.05.2012 | 18 | AP2 | 24.69253 | 59.61823 | 40.4 | 2.47 | 6.82 | 0.7 | 11 |
| 3.05.2012 | 18 | AP2 | 24.69253 | 59.61823 | 93 | 5.1 | 9.38 | 0.43 | 1.7 |
| 3.05.2012 | 18 | AP5 | 24.62698 | 59.68858 | 5.1 | 3.47 | 5.39 | 5.19 | 14.6 |
| 3.05.2012 | 18 | AP5 | 24.62698 | 59.68858 | 40.8 | 2.35 | 6.84 | 0.34 | 11.1 |
| 3.05.2012 | 18 | AP5 | 24.62698 | 59.68858 | 81.8 | 5.24 | 9.64 | 0.19 | 1.1 |
| 9.05.2012 | 19 | AP2 | 24.69253 | 59.61823 | 5.2 | 5.55 | 5.47 | 3.45 | 14.2 |
| 9.05.2012 | 19 | AP2 | 24.69253 | 59.61823 | 40 | 2.44 | 6.76 | 0.75 | 10.6 |
| 9.05.2012 | 19 | AP2 | 24.69253 | 59.61823 | 98 | 5.01 | 9.32 | 0.81 | 1.9 |
| 9.05.2012 | 19 | AP5 | 24.62698 | 59.68858 | 5 | 4.57 | 5.45 | 4.92 | 14.3 |
| 9.05.2012 | 19 | AP5 | 24.62698 | 59.68858 | 40.2 | 2.41 | 6.77 | 0.74 | 10.6 |
| 9.05.2012 | 19 | AP5 | 24.62698 | 59.68858 | 83 | 5.26 | 9.67 | 0.49 | 0.9 |
| 9.05.2012 | 19 | AP8 | 24.56268 | 59.7582 | 5.4 | 4.36 | 5.47 | 4.62 | 13.9 |
| 9.05.2012 | 19 | AP8 | 24.56268 | 59.7582 | 39.9 | 2.1 | 6.65 | 0.43 | 10.8 |
| 9.05.2012 | 19 | AP8 | 24.56268 | 59.7582 | 73.1 | 4.82 | 9.03 | 0.33 | 2.7 |
| 9.05.2012 | 19 | AP11 | 24.50003 | 59.8278 | 5 | 4.59 | 5.48 | 4.58 | 14 |
| 9.05.2012 | 19 | AP11 | 24.50003 | 59.8278 | 50 | 2.62 | 6.95 | 0.85 | 9.9 |
| 9.05.2012 | 19 | AP13 | 24.45807 | 59.8758 | 5.3 | 4.43 | 5.44 | 3.71 | 13.7 |
| 9.05.2012 | 19 | AP13 | 24.45807 | 59.8758 | 29.9 | 3.1 | 5.81 | 1.18 | 12.1 |
| 14.05.2012 | 20 | AP5 | 24.62698 | 59.68858 | 5.2 | 5.35 | 5.53 | 1.53 | 12.5 |
| 14.05.2012 | 20 | AP5 | 24.62698 | 59.68858 | 40.1 | 2.28 | 6.66 | 0.24 | 9.9 |
| 14.05.2012 | 20 | AP5 | 24.62698 | 59.68858 | 82.5 | 5.2 | 9.58 | 0.25 | 0.8 |
| 22.05.2012 | 21 | AP2 | 24.69253 | 59.61823 | 5.4 | 7.49 | 5.65 | 0.72 | 13.2 |
| 22.05.2012 | 21 | AP2 | 24.69253 | 59.61823 | 40.1 | 2.72 | 6.46 | 0.51 | 11.1 |
| 22.05.2012 | 21 | AP2 | 24.69253 | 59.61823 | 89.9 | 5.04 | 9.34 | 0.28 | 1.2 |
| 22.05.2012 | 21 | AP5 | 24.62698 | 59.68858 | 5.1 | 7 | 5.59 | 1.35 | 14 |
| 22.05.2012 | 21 | AP5 | 24.62698 | 59.68858 | 39.8 | 2.69 | 6.56 | 0.58 | 10.9 |
| 22.05.2012 | 21 | AP5 | 24.62698 | 59.68858 | 82.1 | 5.05 | 9.4 | 0.24 | 1.2 |
| 22.05.2012 | 21 | AP8 | 24.56268 | 59.7582 | 5 | 8.2 | 5.66 | 3.55 | 14.4 |
| 22.05.2012 | 21 | AP8 | 24.56268 | 59.7582 | 40.4 | 2.6 | 6.6 | 0.56 | 11.4 |
| 22.05.2012 | 21 | AP8 | 24.56268 | 59.7582 | 75.2 | 4.61 | 8.82 | 0.33 | 3.4 |
| 22.05.2012 | 21 | AP11 | 24.50003 | 59.8278 | 5.1 | 6.33 | 5.4 | 1.6 | 14.6 |
| 22.05.2012 | 21 | AP11 | 24.50003 | 59.8278 | 51.3 | 2.85 | 7 | 0.55 | 9.4 |
| 22.05.2012 | 21 | AP13 | 24.45807 | 59.8758 | 5 | 6.97 | 5.39 | 2.75 | 14.5 |
| 22.05.2012 | 21 | AP13 | 24.45807 | 59.8758 | 31.4 | 3.87 | 5.87 | 0.75 | 11.9 |
| 24.05.2012 | 21 | AP5 | 24.62698 | 59.68858 | 5.2 | 8.18 | 5.66 | 1.27 | 12.8 |
| 24.05.2012 | 21 | AP5 | 24.62698 | 59.68858 | 40.1 | 3.28 | 6.36 | 0.56 | 11.2 |
| 24.05.2012 | 21 | AP5 | 24.62698 | 59.68858 | 83.8 | 4.97 | 9.29 | 0.42 | 1.6 |
| 15.06.2012 | 24 | AP2 | 24.69253 | 59.61823 | 5.1 | 10.74 | 5.37 | 1.09 | 11.6 |
| 15.06.2012 | 24 | AP2 | 24.69253 | 59.61823 | 39.9 | 2.88 | 6.58 | 0.16 | 10.8 |
| 15.06.2012 | 24 | AP2 | 24.69253 | 59.61823 | 93.5 | 5.15 | 9.5 | 0.18 | 0.3 |
| 15.06.2012 | 24 | AP5 | 24.62698 | 59.68858 | 5.2 | 11.1 | 5.36 | 0.81 | 11.6 |
| 15.06.2012 | 24 | AP5 | 24.62698 | 59.68858 | 40.3 | 2.62 | 6.72 | 0.15 | 10.6 |
| 15.06.2012 | 24 | AP5 | 24.62698 | 59.68858 | 81.9 | 5 | 9.27 | 0.16 | 0.4 |
| 15.06.2012 | 24 | AP8 | 24.56268 | 59.7582 | 5.3 | 10.86 | 5.34 | 0.72 | 11.8 |
| 15.06.2012 | 24 | AP8 | 24.56268 | 59.7582 | 40.7 | 2.38 | 6.76 | 0.17 | 10.9 |
| 15.06.2012 | 24 | AP8 | 24.56268 | 59.7582 | 74.3 | 4.48 | 8.65 | 0.2 | 2.8 |
| 15.06.2012 | 24 | AP11 | 24.50003 | 59.8278 | 4.8 | 10.69 | 5.35 | 1.47 | 11.8 |
| 15.06.2012 | 24 | AP11 | 24.50003 | 59.8278 | 39.1 | 2.75 | 6.54 | 0.18 | 10.8 |
| 15.06.2012 | 24 | AP13 | 24.45807 | 59.8758 | 4.9 | 11.33 | 5.49 | 1.45 | 11 |
| 15.06.2012 | 24 | AP13 | 24.45807 | 59.8758 | 31.4 | 3.98 | 6.1 | 0.31 | 11.1 |
| 21.06.2012 | 25 | AP5 | 24.62698 | 59.68858 | 5.1 | 11.09 | 5.42 | 1.85 | 10.6 |
| 21.06.2012 | 25 | AP5 | 24.62698 | 59.68858 | 40 | 3.06 | 6.51 | 0.2 | 10.4 |
| 21.06.2012 | 25 | AP5 | 24.62698 | 59.68858 | 83 | 4.91 | 9.11 | 0.17 | 0.7 |
| 4.07.2012 | 27 | AP2 | 24.69253 | 59.61823 | 5.2 | 14.07 | 5.64 | 2.29 | 9.9 |
| 4.07.2012 | 27 | AP2 | 24.69253 | 59.61823 | 40.5 | 3.72 | 6.61 | 0.5 | 9.9 |
| 4.07.2012 | 27 | AP2 | 24.69253 | 59.61823 | 99 | 4.86 | 9.07 | 0.17 | 0.4 |
| 4.07.2012 | 27 | AP5 | 24.62698 | 59.68858 | 5 | 13.85 | 5.55 | 1.93 | 10.1 |
| 4.07.2012 | 27 | AP5 | 24.62698 | 59.68858 | 40 | 3.01 | 6.74 | 1.32 | 10.3 |
| 4.07.2012 | 27 | AP5 | 24.62698 | 59.68858 | 83 | 4.8 | 9.04 | 0.19 | 0.5 |
| 4.07.2012 | 27 | AP8 | 24.56268 | 59.7582 | 5 | 13.51 | 5.34 | 1.5 | 10.3 |
| 4.07.2012 | 27 | AP8 | 24.56268 | 59.7582 | 39.9 | 2.95 | 6.59 | 0.22 | 9.4 |
| 4.07.2012 | 27 | AP8 | 24.56268 | 59.7582 | 75.2 | 4.57 | 8.73 | 0.22 | 1.9 |
| 4.07.2012 | 27 | AP11 | 24.50003 | 59.8278 | 5.1 | 13.62 | 5.39 | 1.31 | 10.8 |
| 4.07.2012 | 27 | AP11 | 24.50003 | 59.8278 | 48 | 3.15 | 6.93 | 0.29 | 8.4 |
| 4.07.2012 | 27 | AP13 | 24.45807 | 59.8758 | 5.1 | 12.99 | 5.41 | 1.1 | 10.4 |
| 4.07.2012 | 27 | AP13 | 24.45807 | 59.8758 | 32.3 | 4.57 | 6.19 | 1.04 | 9.1 |
| 18.07.2012 | 29 | KERI1 | 25.03944 | 59.70611 | 4.8 | 16.04 | 5.45 | 2.62 | 10 |
| 18.07.2012 | 29 | KERI1 | 25.03944 | 59.70611 | 41 | 4.37 | 6.29 | 0.23 | 9.9 |
| 18.07.2012 | 29 | KERI1 | 25.03944 | 59.70611 | 75 | 4.46 | 8.55 | 0.19 | 2.5 |
| 18.07.2012 | 29 | KERI1 | 25.03944 | 59.70611 | 86.9 | 4.7 | 8.87 | 0.23 | 1 |
| 18.07.2012 | 29 | KERI1 | 25.03944 | 59.70611 | 88.4 | 4.74 | 8.91 | 0.16 | 0.6 |
| 18.07.2012 | 29 | KERI3 | 25.01556 | 59.71639 | 5 | 16.05 | 5.43 | 2.86 | 10 |
| 18.07.2012 | 29 | KERI3 | 25.01556 | 59.71639 | 40.2 | 4.24 | 6.32 | 0.2 | 9.6 |
| 18.07.2012 | 29 | KERI3 | 25.01556 | 59.71639 | 75 | 4.48 | 8.58 | 0.17 | 2.3 |
| 18.07.2012 | 29 | KERI3 | 25.01556 | 59.71639 | 87.1 | 4.68 | 8.81 | 0.31 | 1.1 |
| 18.07.2012 | 29 | KERI3 | 25.01556 | 59.71639 | 104.8 | 4.84 | 9.03 | 0.24 | 0.1 |
| 18.07.2012 | 29 | KERI5 | 24.99694 | 59.72778 | 5.1 | 15.8 | 5.37 | 2.73 | 9.7 |
| 18.07.2012 | 29 | KERI5 | 24.99694 | 59.72778 | 39.6 | 3.79 | 6.32 | 0.25 | 9.8 |
| 18.07.2012 | 29 | KERI5 | 24.99694 | 59.72778 | 70.6 | 4.26 | 8.27 | 0.16 | 3.6 |
| 18.07.2012 | 29 | KERI5 | 24.99694 | 59.72778 | 77.8 | 4.6 | 8.7 | 0.23 | 1.4 |
| 18.07.2012 | 29 | KERI5 | 24.99694 | 59.72778 | 91.9 | 4.72 | 8.84 | 0.16 | 0.7 |
| 20.07.2012 | 29 | AP2 | 24.69253 | 59.61823 | 5.2 | 16.27 | 5.85 | 3.1 | 9.1 |
| 20.07.2012 | 29 | AP2 | 24.69253 | 59.61823 | 40.3 | 15.92 | 5.94 | 0.63 | 8.4 |
| 20.07.2012 | 29 | AP2 | 24.69253 | 59.61823 | 90.1 | 4.79 | 8.98 | 0.17 | 0.3 |
| 20.07.2012 | 29 | AP5 | 24.62698 | 59.68858 | 5 | 16.41 | 5.89 | 3.12 | 9.4 |
| 20.07.2012 | 29 | AP5 | 24.62698 | 59.68858 | 40.7 | 10.72 | 5.81 | 0.4 | 8.9 |
| 20.07.2012 | 29 | AP5 | 24.62698 | 59.68858 | 82.5 | 4.61 | 8.71 | 0.21 | 1.5 |
| 20.07.2012 | 29 | AP8 | 24.56268 | 59.7582 | 5 | 16.32 | 5.77 | 3.91 | 9.7 |
| 20.07.2012 | 29 | AP8 | 24.56268 | 59.7582 | 39.9 | 4.37 | 6.29 | 0.2 | 9.6 |
| 20.07.2012 | 29 | AP8 | 24.56268 | 59.7582 | 75 | 3.84 | 7.89 | 0.16 | 4.9 |
| 20.07.2012 | 29 | AP11 | 24.50003 | 59.8278 | 4.9 | 16.21 | 5.44 | 3.19 | 9.8 |
| 20.07.2012 | 29 | AP11 | 24.50003 | 59.8278 | 45 | 3.15 | 6.76 | 0.4 | 9.2 |
| 20.07.2012 | 29 | AP13 | 24.45807 | 59.8758 | 5 | 15.73 | 5.61 | 2.3 | 9.7 |
| 20.07.2012 | 29 | AP13 | 24.45807 | 59.8758 | 37.4 | 3.46 | 6.47 | 0.47 | 8.9 |
| 31.07.2012 | 31 | KERI1 | 25.03944 | 59.70611 | 5.1 | 18.2 | 5.85 | 3.1 | 9.1 |
| 31.07.2012 | 31 | KERI1 | 25.03944 | 59.70611 | 40 | 4.83 | 6.01 | 0.21 | 9.5 |
| 31.07.2012 | 31 | KERI1 | 25.03944 | 59.70611 | 75 | 3.97 | 7.73 | 0.22 | 4.7 |
| 31.07.2012 | 31 | KERI1 | 25.03944 | 59.70611 | 80 | 4.03 | 7.8 | 0.16 | 4.2 |
| 31.07.2012 | 31 | KERI1 | 25.03944 | 59.70611 | 85.2 | 4.37 | 8.29 | 0.17 | 2.4 |
| 31.07.2012 | 31 | KERI3 | 25.01556 | 59.71639 | 5 | 17.9 | 5.86 | 4 | 9.3 |
| 31.07.2012 | 31 | KERI3 | 25.01556 | 59.71639 | 39.8 | 5.79 | 5.99 | 0.25 | 9.2 |
| 31.07.2012 | 31 | KERI3 | 25.01556 | 59.71639 | 85 | 4.33 | 8.27 | 0.17 | 2.4 |
| 31.07.2012 | 31 | KERI3 | 25.01556 | 59.71639 | 95 | 4.63 | 8.73 | 0.16 | 0.7 |
| 31.07.2012 | 31 | KERI3 | 25.01556 | 59.71639 | 104.8 | 4.76 | 8.89 | 0.19 | 0.3 |
| 31.07.2012 | 31 | KERI5 | 24.99694 | 59.72778 | 5.1 | 18.27 | 5.86 | 2.88 | 9.2 |
| 31.07.2012 | 31 | KERI5 | 24.99694 | 59.72778 | 40 | 5.29 | 5.95 | 0.26 | 9.5 |
| 31.07.2012 | 31 | KERI5 | 24.99694 | 59.72778 | 79.9 | 4.15 | 7.99 | 0.17 | 3.6 |
| 31.07.2012 | 31 | KERI5 | 24.99694 | 59.72778 | 87.2 | 4.57 | 8.65 | 0.16 | 1 |
| 31.07.2012 | 31 | KERI5 | 24.99694 | 59.72778 | 95.1 | 4.66 | 8.76 | 0.16 | 0.6 |
| 13.08.2012 | 33 | AP2 | 24.69253 | 59.61823 | 4.9 | 17.87 | 5.8 | 2.89 | 8.3 |
| 13.08.2012 | 33 | AP2 | 24.69253 | 59.61823 | 40.4 | 4.32 | 6.62 | 0.17 | 8.3 |
| 13.08.2012 | 33 | AP2 | 24.69253 | 59.61823 | 99.8 | 4.51 | 8.56 | 0.16 | 0.8 |
| 13.08.2012 | 33 | AP5 | 24.62698 | 59.68858 | 4.8 | 18.12 | 5.92 | 3.09 | 8.1 |
| 13.08.2012 | 33 | AP5 | 24.62698 | 59.68858 | 40.3 | 4.15 | 6.33 | 0.19 | 8.5 |
| 13.08.2012 | 33 | AP5 | 24.62698 | 59.68858 | 82 | 4.45 | 8.53 | 0.17 | 1.4 |
| 13.08.2012 | 33 | AP8 | 24.56268 | 59.7582 | 4.8 | 18.01 | 5.82 | 3.64 | 8.5 |
| 13.08.2012 | 33 | AP8 | 24.56268 | 59.7582 | 40 | 4.93 | 5.86 | 0.21 | 8.7 |
| 13.08.2012 | 33 | AP8 | 24.56268 | 59.7582 | 74.1 | 4.28 | 8.27 | 0.16 | 2.2 |
| 13.08.2012 | 33 | AP11 | 24.50003 | 59.8278 | 5.1 | 18.18 | 5.84 | 4.39 | 8.6 |
| 13.08.2012 | 33 | AP11 | 24.50003 | 59.8278 | 49.3 | 5.47 | 6.01 | 0.22 | 8.4 |
| 13.08.2012 | 33 | AP13 | 24.45807 | 59.8758 | 4.6 | 18.19 | 5.8 | 3.25 | 8.6 |
| 13.08.2012 | 33 | AP13 | 24.45807 | 59.8758 | 30.1 | 9.32 | 5.82 | 0.24 | 8.2 |
| 4.09.2012 | 36 | KERI1 | 25.03944 | 59.70611 | 5 | 16.48 | 5.46 | 1.66 | 8.5 |
| 4.09.2012 | 36 | KERI1 | 25.03944 | 59.70611 | 40.4 | 5.44 | 6.12 | 0.21 | 8.2 |
| 4.09.2012 | 36 | KERI1 | 25.03944 | 59.70611 | 54.8 | 3.67 | 6.84 | 0.17 | 7.6 |
| 4.09.2012 | 36 | KERI1 | 25.03944 | 59.70611 | 69.9 | 4.26 | 8.33 | 0.19 | 2 |
| 4.09.2012 | 36 | KERI1 | 25.03944 | 59.70611 | 83.6 | 4.75 | 9.07 | 0.18 | 0.3 |
| 4.09.2012 | 36 | KERI3 | 25.01556 | 59.71639 | 4.5 | 16.41 | 5.49 | 1.46 | 8.2 |
| 4.09.2012 | 36 | KERI3 | 25.01556 | 59.71639 | 40 | 5.31 | 6.17 | 0.19 | 8.1 |
| 4.09.2012 | 36 | KERI3 | 25.01556 | 59.71639 | 62.1 | 3.76 | 7.43 | 0.17 | 5.3 |
| 4.09.2012 | 36 | KERI3 | 25.01556 | 59.71639 | 84.9 | 4.79 | 9.13 | 0.18 | 0.1 |
| 4.09.2012 | 36 | KERI3 | 25.01556 | 59.71639 | 105.7 | 4.87 | 9.24 | 0.19 | 0.2 |
| 4.09.2012 | 36 | KERI5 | 24.99694 | 59.72778 | 4.6 | 16.48 | 5.47 | 2.11 | 8.2 |
| 4.09.2012 | 36 | KERI5 | 24.99694 | 59.72778 | 40.1 | 5.04 | 6.26 | 0.18 | 8.2 |
| 4.09.2012 | 36 | KERI5 | 24.99694 | 59.72778 | 80.6 | 4.62 | 8.83 | 0.18 | 0.1 |
| 4.09.2012 | 36 | KERI5 | 24.99694 | 59.72778 | 96.3 | 4.82 | 9.18 | 0.19 | 0.3 |
| 9.10.2012 | 41 | KERI1 | 25.03944 | 59.70611 | 4.9 | 12.22 | 5.7 | 1.01 | 10 |
| 9.10.2012 | 41 | KERI1 | 25.03944 | 59.70611 | 39.3 | 12.74 | 5.96 | 0.67 | 9.7 |
| 9.10.2012 | 41 | KERI1 | 25.03944 | 59.70611 | 54.1 | 4.86 | 6.19 | 0.21 | 7.3 |
| 9.10.2012 | 41 | KERI1 | 25.03944 | 59.70611 | 74.5 | 3.91 | 7.3 | 0.18 | 4.3 |
| 9.10.2012 | 41 | KERI1 | 25.03944 | 59.70611 | 86.9 | 4.19 | 7.96 | 0.2 | 1.9 |
| 9.10.2012 | 41 | KERI3 | 25.01556 | 59.71639 | 4.6 | 12.19 | 5.69 | 1.02 | 10.1 |
| 9.10.2012 | 41 | KERI3 | 25.01556 | 59.71639 | 40 | 12.85 | 5.99 | 0.82 | 9.9 |
| 9.10.2012 | 41 | KERI3 | 25.01556 | 59.71639 | 53.1 | 6.55 | 6.12 | 0.22 | 7.8 |
| 9.10.2012 | 41 | KERI3 | 25.01556 | 59.71639 | 73.4 | 3.88 | 7.3 | 0.18 | 4 |
| 9.10.2012 | 41 | KERI3 | 25.01556 | 59.71639 | 105.8 | 4.36 | 8.31 | 0.18 | 0.4 |
| 9.10.2012 | 41 | KERI5 | 24.99694 | 59.72778 | 5.1 | 12.15 | 5.69 | 1.27 | 10 |
| 9.10.2012 | 41 | KERI5 | 24.99694 | 59.72778 | 39.7 | 12.19 | 5.71 | 1.01 | 9.5 |
| 9.10.2012 | 41 | KERI5 | 24.99694 | 59.72778 | 55.3 | 5.24 | 6.27 | 0.21 | 7.5 |
| 9.10.2012 | 41 | KERI5 | 24.99694 | 59.72778 | 72.1 | 3.88 | 7.23 | 0.17 | 4.6 |
| 9.10.2012 | 41 | KERI5 | 24.99694 | 59.72778 | 93.7 | 4.22 | 8.07 | 0.19 | 1.3 |
