# Supplementary material for: Near-Bottom Hypoxia Impacts Dynamics of Bacterioplankton Assemblage throughout Water Column of the Gulf of Finland (Baltic Sea)
Source: PLoS One. 2016 May 23;11(5):e0156147. doi: 10.1371/journal.pone.0156147 (PMC4877108; doi:10.1371/journal.pone.0156147)
Supplement: S2 Table — (DOCX) [file pone.0156147.s005.docx]

|  | | | | | | |
| --- | --- | --- | --- | --- | --- | --- |
| **Date** | **Station** | **Depth (m)** | **Sequences** | **OTUs observed** | **Chao1** | **ACE** |
| 8.11.2011 | AP2 | 4.9 | 2507 | 164 | 235 | 262 |
| 8.11.2011 | AP2 | 94.6 | 6844 | 272 | 483 | 466 |
| 8.11.2011 | AP5 | 4.8 | 6110 | 220 | 363 | 343 |
| 8.11.2011 | AP5 | 82.3 | 4355 | 238 | 498 | 460 |
| 4.04.2012 | AP2 | 4.8 | 4371 | 257 | 541 | 482 |
| 4.04.2012 | AP2 | 40.3 | 10632 | 280 | 672 | 568 |
| 4.04.2012 | AP2 | 90.9 | 5603 | 163 | 244 | 251 |
| 4.04.2012 | AP5 | 5.5 | 9805 | 280 | 588 | 499 |
| 4.04.2012 | AP5 | 40.1 | 4878 | 202 | 463 | 455 |
| 4.04.2012 | AP5 | 82.6 | 3972 | 154 | 258 | 258 |
| 4.04.2012 | AP8 | 5 | 2616 | 242 | 526 | 538 |
| 4.04.2012 | AP8 | 39.7 | 7663 | 188 | 359 | 356 |
| 4.04.2012 | AP8 | 78.3 | 3788 | 180 | 284 | 297 |
| 4.04.2012 | AP11 | 4.8 | 3777 | 153 | 255 | 285 |
| 4.04.2012 | AP11 | 53.1 | 3678 | 212 | 341 | 345 |
| 4.04.2012 | AP13 | 4.5 | 4033 | 225 | 455 | 424 |
| 4.04.2012 | AP13 | 31.1 | 2489 | 197 | 348 | 341 |
| 23.04.2012 | AP2 | 5.2 | 3167 | 192 | 390 | 377 |
| 23.04.2012 | AP2 | 40.7 | 3225 | 132 | 227 | 253 |
| 23.04.2012 | AP2 | 85.5 | 5397 | 265 | 428 | 457 |
| 23.04.2012 | AP5 | 41.2 | 3779 | 255 | 595 | 565 |
| 23.04.2012 | AP5 | 83 | 4700 | 158 | 221 | 236 |
| 23.04.2012 | AP8 | 5.5 | 3111 | 232 | 427 | 465 |
| 23.04.2012 | AP8 | 40.3 | 6945 | 120 | 187 | 202 |
| 23.04.2012 | AP8 | 74.5 | 10429 | 159 | 258 | 257 |
| 23.04.2012 | AP11 | 5 | 4540 | 132 | 250 | 220 |
| 23.04.2012 | AP11 | 52 | 4005 | 228 | 363 | 374 |
| 23.04.2012 | AP13 | 5.1 | 3407 | 195 | 354 | 350 |
| 23.04.2012 | AP13 | 31 | 8318 | 158 | 267 | 258 |
| 3.05.2012 | AP2 | 5.1 | 3855 | 198 | 327 | 323 |
| 3.05.2012 | AP2 | 40.4 | 3898 | 209 | 384 | 415 |
| 3.05.2012 | AP2 | 93 | 7372 | 147 | 274 | 239 |
| 3.05.2012 | AP5 | 5.1 | 4037 | 110 | 160 | 183 |
| 3.05.2012 | AP5 | 40.8 | 2650 | 178 | 353 | 392 |
| 3.05.2012 | AP5 | 81.8 | 6424 | 207 | 315 | 344 |
| 9.05.2012 | AP2 | 5.2 | 4839 | 205 | 343 | 360 |
| 9.05.2012 | AP2 | 40 | 4438 | 193 | 328 | 316 |
| 9.05.2012 | AP2 | 98 | 5008 | 144 | 210 | 229 |
| 9.05.2012 | AP5 | 5 | 3895 | 181 | 347 | 300 |
| 9.05.2012 | AP5 | 40.2 | 2858 | 220 | 345 | 392 |
| 9.05.2012 | AP5 | 83 | 4459 | 127 | 175 | 175 |
| 9.05.2012 | AP8 | 5.4 | 3121 | 203 | 382 | 401 |
| 9.05.2012 | AP8 | 39.9 | 6678 | 126 | 171 | 172 |
| 9.05.2012 | AP8 | 73.1 | 6217 | 114 | 159 | 174 |
| 9.05.2012 | AP11 | 5 | 5628 | 132 | 207 | 208 |
| 9.05.2012 | AP11 | 50 | 4410 | 149 | 228 | 221 |
| 9.05.2012 | AP13 | 5.3 | 3561 | 188 | 302 | 334 |
| 9.05.2012 | AP13 | 29.9 | 3141 | 172 | 272 | 291 |
| 14.05.2012 | AP5 | 5.2 | 3461 | 161 | 319 | 322 |
| 14.05.2012 | AP5 | 40.1 | 6206 | 147 | 253 | 236 |
| 14.05.2012 | AP5 | 82.5 | 3420 | 194 | 348 | 371 |
| 22.05.2012 | AP2 | 5.4 | 3068 | 169 | 378 | 397 |
| 22.05.2012 | AP2 | 40.1 | 2786 | 137 | 191 | 198 |
| 22.05.2012 | AP2 | 89.9 | 7897 | 206 | 312 | 332 |
| 22.05.2012 | AP5 | 5.1 | 2581 | 142 | 223 | 254 |
| 22.05.2012 | AP5 | 39.8 | 5681 | 134 | 306 | 225 |
| 22.05.2012 | AP5 | 82.1 | 3606 | 215 | 328 | 374 |
| 22.05.2012 | AP8 | 5 | 3438 | 116 | 148 | 157 |
| 22.05.2012 | AP8 | 40.4 | 3493 | 144 | 266 | 235 |
| 22.05.2012 | AP8 | 75.2 | 2561 | 120 | 193 | 183 |
| 22.05.2012 | AP11 | 5.1 | 2204 | 127 | 166 | 193 |
| 22.05.2012 | AP11 | 51.3 | 4015 | 174 | 320 | 342 |
| 22.05.2012 | AP13 | 5 | 4533 | 135 | 233 | 237 |
| 22.05.2012 | AP13 | 31.4 | 6214 | 162 | 304 | 277 |
| 24.05.2012 | AP5 | 5.2 | 4898 | 192 | 402 | 449 |
| 24.05.2012 | AP5 | 40.1 | 4694 | 117 | 208 | 212 |
| 24.05.2012 | AP5 | 83.8 | 3686 | 154 | 253 | 269 |
| 15.06.2012 | AP2 | 5.1 | 3830 | 185 | 384 | 367 |
| 15.06.2012 | AP2 | 39.9 | 6239 | 142 | 291 | 258 |
| 15.06.2012 | AP2 | 93.5 | 4371 | 113 | 185 | 203 |
| 15.06.2012 | AP5 | 5.2 | 3219 | 159 | 313 | 301 |
| 15.06.2012 | AP5 | 40.3 | 5912 | 124 | 205 | 180 |
| 15.06.2012 | AP5 | 81.9 | 3904 | 128 | 220 | 203 |
| 15.06.2012 | AP8 | 5.3 | 4173 | 121 | 204 | 206 |
| 15.06.2012 | AP8 | 40.7 | 2153 | 151 | 340 | 245 |
| 15.06.2012 | AP8 | 74.3 | 1909 | 84 | 142 | 129 |
| 15.06.2012 | AP11 | 4.8 | 5641 | 139 | 256 | 283 |
| 15.06.2012 | AP11 | 39.1 | 5556 | 102 | 166 | 165 |
| 15.06.2012 | AP13 | 4.9 | 3786 | 185 | 300 | 344 |
| 15.06.2012 | AP13 | 31.4 | 6625 | 155 | 239 | 253 |
| 21.06.2012 | AP5 | 5.1 | 3475 | 95 | 145 | 131 |
| 21.06.2012 | AP5 | 40 | 6665 | 144 | 205 | 213 |
| 21.06.2012 | AP5 | 83 | 7183 | 157 | 319 | 338 |
| 4.07.2012 | AP2 | 5.2 | 4235 | 174 | 269 | 289 |
| 4.07.2012 | AP2 | 40.5 | 4218 | 119 | 202 | 202 |
| 4.07.2012 | AP2 | 99 | 6045 | 213 | 419 | 403 |
| 4.07.2012 | AP5 | 5 | 5628 | 133 | 195 | 218 |
| 4.07.2012 | AP5 | 40 | 6870 | 211 | 491 | 440 |
| 4.07.2012 | AP5 | 83 | 5915 | 140 | 206 | 227 |
| 4.07.2012 | AP8 | 5 | 3293 | 142 | 258 | 270 |
| 4.07.2012 | AP8 | 39.9 | 4614 | 196 | 386 | 366 |
| 4.07.2012 | AP8 | 75.2 | 5791 | 164 | 241 | 268 |
| 4.07.2012 | AP11 | 5.1 | 4820 | 133 | 288 | 331 |
| 4.07.2012 | AP11 | 48 | 5437 | 150 | 217 | 238 |
| 4.07.2012 | AP13 | 5.1 | 4461 | 159 | 305 | 273 |
| 4.07.2012 | AP13 | 32.3 | 3257 | 164 | 300 | 299 |
| 18.07.2012 | KERI1 | 4.8 | 2856 | 121 | 195 | 189 |
| 18.07.2012 | KERI1 | 41 | 5806 | 172 | 395 | 375 |
| 18.07.2012 | KERI1 | 75 | 5551 | 148 | 260 | 271 |
| 18.07.2012 | KERI1 | 86.9 | 2824 | 148 | 258 | 301 |
| 18.07.2012 | KERI1 | 88.4 | 2708 | 154 | 252 | 253 |
| 18.07.2012 | KERI3 | 5 | 1840 | 131 | 224 | 249 |
| 18.07.2012 | KERI3 | 40.2 | 3783 | 149 | 271 | 243 |
| 18.07.2012 | KERI3 | 75 | 3185 | 135 | 253 | 243 |
| 18.07.2012 | KERI3 | 87.1 | 3779 | 139 | 257 | 291 |
| 18.07.2012 | KERI3 | 104.8 | 3051 | 99 | 145 | 149 |
| 18.07.2012 | KERI5 | 5.1 | 4522 | 189 | 284 | 285 |
| 18.07.2012 | KERI5 | 39.6 | 4802 | 143 | 230 | 260 |
| 18.07.2012 | KERI5 | 70.6 | 2515 | 105 | 167 | 170 |
| 18.07.2012 | KERI5 | 77.8 | 7039 | 174 | 287 | 315 |
| 18.07.2012 | KERI5 | 91.9 | 2693 | 169 | 348 | 326 |
| 20.07.2012 | AP2 | 5.2 | 8095 | 121 | 219 | 191 |
| 20.07.2012 | AP2 | 40.3 | 12369 | 323 | 592 | 656 |
| 20.07.2012 | AP2 | 90.1 | 6141 | 153 | 246 | 262 |
| 20.07.2012 | AP5 | 5 | 4065 | 196 | 321 | 369 |
| 20.07.2012 | AP5 | 40.7 | 3437 | 153 | 266 | 263 |
| 20.07.2012 | AP5 | 82.5 | 7480 | 168 | 245 | 255 |
| 20.07.2012 | AP8 | 5 | 6197 | 240 | 490 | 512 |
| 20.07.2012 | AP8 | 39.9 | 3375 | 172 | 324 | 346 |
| 20.07.2012 | AP8 | 75 | 3842 | 155 | 298 | 296 |
| 20.07.2012 | AP11 | 4.9 | 3508 | 146 | 264 | 275 |
| 20.07.2012 | AP11 | 45 | 4117 | 146 | 186 | 203 |
| 20.07.2012 | AP13 | 5 | 4824 | 162 | 266 | 287 |
| 20.07.2012 | AP13 | 37.4 | 3558 | 187 | 422 | 393 |
| 31.07.2012 | KERI1 | 5.1 | 5483 | 171 | 279 | 295 |
| 31.07.2012 | KERI1 | 40 | 3566 | 209 | 389 | 390 |
| 31.07.2012 | KERI1 | 75 | 3036 | 126 | 204 | 225 |
| 31.07.2012 | KERI1 | 80 | 2098 | 118 | 195 | 224 |
| 31.07.2012 | KERI1 | 85.2 | 4849 | 215 | 382 | 381 |
| 31.07.2012 | KERI3 | 5 | 4892 | 201 | 379 | 406 |
| 31.07.2012 | KERI3 | 39.8 | 6224 | 159 | 231 | 256 |
| 31.07.2012 | KERI3 | 85 | 6481 | 197 | 347 | 300 |
| 31.07.2012 | KERI3 | 95 | 2926 | 178 | 345 | 391 |
| 31.07.2012 | KERI3 | 104.8 | 6711 | 181 | 289 | 281 |
| 31.07.2012 | KERI5 | 5.1 | 7398 | 265 | 438 | 422 |
| 31.07.2012 | KERI5 | 40 | 4023 | 143 | 270 | 265 |
| 31.07.2012 | KERI5 | 79.9 | 2367 | 154 | 214 | 225 |
| 31.07.2012 | KERI5 | 87.2 | 4603 | 195 | 315 | 309 |
| 31.07.2012 | KERI5 | 95.1 | 2154 | 182 | 310 | 309 |
| 13.08.2012 | AP2 | 4.9 | 2678 | 200 | 451 | 471 |
| 13.08.2012 | AP2 | 40.4 | 8711 | 199 | 360 | 372 |
| 13.08.2012 | AP2 | 99.8 | 2285 | 203 | 327 | 384 |
| 13.08.2012 | AP5 | 4.8 | 3168 | 131 | 241 | 217 |
| 13.08.2012 | AP5 | 40.3 | 6780 | 270 | 443 | 480 |
| 13.08.2012 | AP5 | 82 | 6394 | 261 | 606 | 606 |
| 13.08.2012 | AP8 | 4.8 | 4068 | 207 | 401 | 368 |
| 13.08.2012 | AP8 | 40 | 4733 | 262 | 637 | 642 |
| 13.08.2012 | AP8 | 74.1 | 3959 | 175 | 249 | 264 |
| 13.08.2012 | AP11 | 5.1 | 4005 | 151 | 330 | 361 |
| 13.08.2012 | AP11 | 49.3 | 2569 | 144 | 227 | 234 |
| 13.08.2012 | AP13 | 4.6 | 3171 | 155 | 278 | 272 |
| 13.08.2012 | AP13 | 30.1 | 4970 | 197 | 287 | 285 |
| 4.09.2012 | KERI1 | 5 | 6889 | 280 | 433 | 389 |
| 4.09.2012 | KERI1 | 40.4 | 5816 | 215 | 376 | 400 |
| 4.09.2012 | KERI1 | 54.8 | 3628 | 205 | 423 | 308 |
| 4.09.2012 | KERI1 | 69.9 | 5116 | 255 | 396 | 371 |
| 4.09.2012 | KERI1 | 83.6 | 5481 | 227 | 492 | 436 |
| 4.09.2012 | KERI3 | 4.5 | 4294 | 172 | 442 | 445 |
| 4.09.2012 | KERI3 | 40 | 6041 | 261 | 434 | 418 |
| 4.09.2012 | KERI3 | 62.1 | 3644 | 177 | 290 | 267 |
| 4.09.2012 | KERI3 | 84.9 | 3581 | 157 | 226 | 254 |
| 4.09.2012 | KERI3 | 105.7 | 3695 | 219 | 450 | 434 |
| 4.09.2012 | KERI5 | 4.6 | 15417 | 749 | 2667 | 2603 |
| 4.09.2012 | KERI5 | 40.1 | 6683 | 385 | 679 | 686 |
| 4.09.2012 | KERI5 | 80.6 | 6966 | 202 | 296 | 335 |
| 4.09.2012 | KERI5 | 96.3 | 5229 | 307 | 537 | 573 |
| 9.10.2012 | KERI1 | 4.9 | 11473 | 271 | 456 | 443 |
| 9.10.2012 | KERI1 | 39.3 | 23720 | 217 | 327 | 320 |
| 9.10.2012 | KERI1 | 54.1 | 9600 | 234 | 495 | 461 |
| 9.10.2012 | KERI1 | 74.5 | 8139 | 300 | 602 | 629 |
| 9.10.2012 | KERI1 | 86.9 | 7834 | 328 | 544 | 539 |
| 9.10.2012 | KERI3 | 4.6 | 22629 | 630 | 1237 | 1271 |
| 9.10.2012 | KERI3 | 40 | 11177 | 220 | 326 | 363 |
| 9.10.2012 | KERI3 | 53.1 | 3679 | 148 | 283 | 277 |
| 9.10.2012 | KERI3 | 73.4 | 11567 | 397 | 836 | 811 |
| 9.10.2012 | KERI3 | 105.8 | 11362 | 304 | 468 | 513 |
| 9.10.2012 | KERI5 | 5.1 | 5943 | 213 | 302 | 342 |
| 9.10.2012 | KERI5 | 39.7 | 10527 | 296 | 526 | 579 |
| 9.10.2012 | KERI5 | 55.3 | 13835 | 288 | 566 | 538 |
| 9.10.2012 | KERI5 | 72.1 | 11013 | 339 | 657 | 642 |
| 9.10.2012 | KERI5 | 93.7 | 11489 | 376 | 730 | 631 |
